# EF-P Dependent Pauses Integrate Proximal and Distal Signals during Translation

**DOI:** 10.1371/journal.pgen.1004553

**Published:** 2014-08-21

**Authors:** Sara Elgamal, Assaf Katz, Steven J. Hersch, David Newsom, Peter White, William Wiley Navarre, Michael Ibba

**Affiliations:** 1 Department of Microbiology and The Center for RNA Biology, Ohio State University, Columbus, Ohio, United States of America; 2 Programa de Biología Celular y Molecular, ICBM, Facultad de Medicina, Universidad de Chile, Santiago, Chile; 3 Department of Molecular Genetics, University of Toronto, Toronto, Ontario, Canada; 4 Center for Microbial Pathogenesis, The Research Institute at Nationwide Children's Hospital, Columbus, Ohio, United States of America; 5 Department of Pediatrics, Ohio State University, Columbus, Ohio, United States of America; University of California Santa Barbara, United States of America

## Abstract

Elongation factor P (EF-P) is required for the efficient synthesis of proteins with stretches of consecutive prolines and other motifs that would otherwise lead to ribosome pausing. However, previous reports also demonstrated that levels of most diprolyl-containing proteins are not altered by the deletion of *efp*. To define the particular sequences that trigger ribosome stalling at diprolyl (PPX) motifs, we used ribosome profiling to monitor global ribosome occupancy in *Escherichia coli* strains lacking EF-P. Only 2.8% of PPX motifs caused significant ribosomal pausing in the Δ*efp* strain, with up to a 45-fold increase in ribosome density observed at the pausing site. The unexpectedly low fraction of PPX motifs that produce a pause in translation led us to investigate the possible role of sequences upstream of PPX. Our data indicate that EF-P dependent pauses are strongly affected by sequences upstream of the PPX pattern. We found that residues as far as 3 codons upstream of the ribosomal peptidyl-tRNA site had a dramatic effect on whether or not a particular PPX motif triggered a ribosomal pause, while internal Shine Dalgarno sequences upstream of the motif had no effect on EF-P dependent translation efficiency. Increased ribosome occupancy at particular stall sites did not reliably correlate with a decrease in total protein levels, suggesting that in many cases other factors compensate for the potentially deleterious effects of stalling on protein synthesis. These findings indicate that the ability of a given PPX motif to initiate an EF-P-alleviated stall is strongly influenced by its local context, and that other indirect post-transcriptional effects determine the influence of such stalls on protein levels within the cell.

## Author Summary

Elongation factor P (EF-P) is a well-conserved bacterial protein. Although it can enhance protein synthesis *in vitro*, it is generally regarded as an ancillary factor required for robust translation of transcripts with stretches of consecutive prolines. In this work we performed ribosome profiling to better understand the role of EF-P during translation. Our data confirmed that translational effects due to lack of EF-P are mainly confined to PPX–encoding genes. Wide variations in EF-P dependent translation of these PPXs led us to investigate the effect of sequences upstream of diproline-containing motifs. We found that amino acids encoded upstream of PPX play a key role in EF-P-dependent translation. Finally, comparison of ribosome profiling data to existing proteomic data indicates that although many PPX-containing patterns have increased ribosome occupancies, this does not necessarily lead to altered protein levels. Taken together these data show a direct role for EF-P during synthesis of PPX motifs, and indirect effects on other post-transcriptional regulators of gene expression.

## Introduction

During protein synthesis each amino acid is detached from an aminoacyl-tRNA and incorporated into the nascent peptide. Although the basic peptidyl transfer reaction is the same for all amino acids, the speed of incorporation is not uniform. It is affected by several factors including the abundance of each individual aminoacyl-tRNA, the structure of the incorporated amino acid, and structural features of the mRNA and the nascent peptide. For example, mRNA sequences upstream of the peptidyl-tRNA site (P site) codon that interact with the anti Shine Dalgarno sequence (aSD) from 16S rRNA [1] or regions from the nascent peptide that interact with the ribosome exit tunnel, have been shown to slow translation [2]–[4].

Decreasing the speed of translation, or even pausing it, can have important roles in protein synthesis. For instance, sequence context dependent pausing during translation of *secM* is known to regulate synthesis of the membrane protein SecA [5]. More broadly, changes in translation speed can affect co-translational folding of proteins, controlling not only the fraction of active protein [6], but potentially also providing new functionality through alternative folds [7]. Although in these cases translation pausing has beneficial physiological roles, in other cases it could be detrimental if it significantly decreases the efficiency of protein synthesis. Accordingly, patterns that induce ribosome pausing are often excluded from coding regions [1], [4]. Exceptions to this include PPP and PPG sequences. This is most probably due to the presence of elongation factor P (EF-P) [4], a protein that has been recently shown to prevent the pauses produced by these and other sequences, most of which contain a PP motif [8]–[11]. It has been described that in the absence of EF-P, mRNA coding for PPG will pause with Gly-tRNA^Gly^ located at the A site of the ribosome and peptidyl-tRNA^Pro^ at the P site [8]. A similar effect has been reported for PPP sequences that pause with the second Pro at the P site [4].

EF-P is a remarkable example of molecular mimicry. The protein is similar in shape and size to a tRNA and interacts with the ribosome via the exit (E) site on the 30S subunit and the peptidyl-transferase center (PTC) of the 50S subunit (PDB 1UEB, [12]–[14]). Presumably during a PPX-induced stall in translation the E-site tRNA is ejected, allowing EF-P access to the ribosome where its N-terminal domain can insert into the PTC to re-initiate synthesis. EF-P activity requires post-translational modification by the addition of (*R*)-β-lysine to Lys34 (*Escherichia coli* numbering) in a reaction that is catalyzed by PoxA, a paralog of the catalytic domain of lysyl-tRNA synthetase [13], [15]. Further hydroxylation of EF-P Lys34 has also been observed, but the role of EF-P hydroxylation is unclear as no adverse fitness effects have been found in its absence [11], [16], [17]. EF-P is homologous to the eukaryotic eIF5A protein, which is post-translationally modified at an analogous lysyl residue and has also been shown to stimulate the synthesis of proteins containing polyproline motifs [18], [19].

While PPP, PPG and some other PPX sequences (where X represents any of the 20 proteinogenic amino acids) usually trigger pausing *in vitro* in the absence of EF-P, this is not always true *in vivo*. We and others observed that the total levels of most proteins containing PPP or PPG sequences are not affected by the loss of EF-P in either *E. coli* or *Salmonella* [10], [11]. For example the *atpA* and *atpD* genes both encode a PPG motif, but proteomic analysis demonstrates that only AtpD levels are affected by *efp* deletion [10], [11]. Proteomic approaches are sensitive to protein degradation and synthesis, making it difficult to distinguish the underlying cause of changes in protein levels [20], which could also result from ribosome pausing or indirect changes in regulatory proteins upon *efp* deletion. To address how EF-P regulates protein levels in the cell, here we apply ribosome profiling to globally identify the set of sequences that trigger ribosomal pausing in the absence of EF-P. We analyzed this dataset in conjunction with earlier proteomic data to define the specific features that differentiate the PPX sequences that produce pausing during translation from those that do not. Our data indicate that pausing-potential is largely influenced by the local context of the PPX pattern, and that specific amino acids upstream of the PPX motif can modulate whether or not a particular A site residue can trigger a stall.

## Results

### Ribosome profiling of wild type, Δefp and Δefp complemented *E. coli* strains

Ribosome profiling, or ribo-seq, is a genome-wide, quantitative analysis of ribosome occupancy *in vivo* by nuclease footprinting and deep sequencing. It can map the precise position and density of ribosomes on transcripts, and provides a direct readout of which sequences cause stalling [21]. Ribo-seq was performed for wild type *E. coli*, Δ*efp* and Δ*efp* complemented strains (Δ*efp* pEF-P, complemented with a plasmid expressing *efp*). Cells were harvested at mid-log phase and collected by rapid filtration followed by rapid freezing in liquid nitrogen [22]. Nuclease-treated (footprints) and untreated (total) mRNA samples were processed for each of the strains. The correlation between the two biological replicates for each strain was between 96–98% (Fig. S1).

### Increased ribosome occupancy of diprolyl-encoding messages

**Figure 1:**
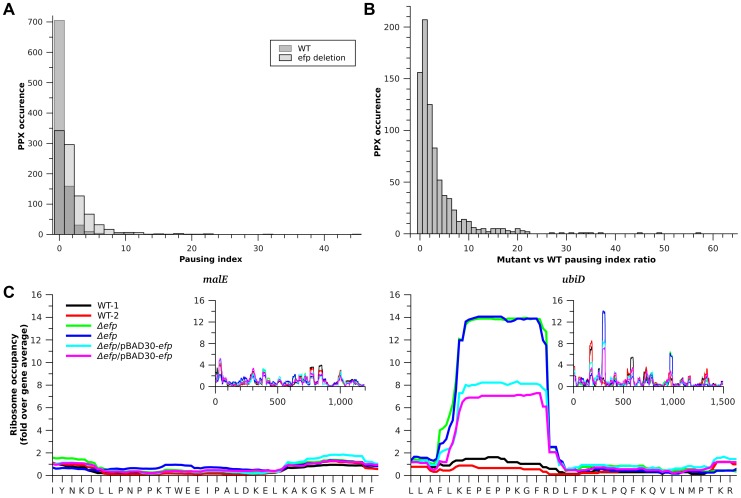
Translation pausing at PPX sequences. **A**) Histogram of pausing index of PPX sequences in WT (dark gray) and Δ*efp* strains (light gray). **B**) Histogram of changes in pausing index of PPX after *efp* deletion. **C**) Example of two genes bearing different ribosome occupancies for a similar PPX sequence. Left, *malE* contains a PNPPK sequence that does not produce translation pausing and right *ubiD* contains a PEPPK sequence that produces a pause in translation. Insets show the ribosome occupancies for the full genes. Black and red are used for the two WT strain replicates, green and blue for the Δ*efp* replicates, while cyan and magenta are used for the complemented strain replicates. Values in the figure are normalized to the corresponding gene ribosome occupancy average.

EF-P prevents translational pausing during synthesis of some polyproline-containing proteins [8]–[11]. *E coli* has over 2000 PPX motifs encoded in its genome, of which 913 had significant reads in our ribo-seq data (i.e. with a coverage of at least 3 sequencing reads per codon). Translational pauses cause the accumulation of ribosomes at the pausing site and increased density in ribosome profiling (i.e. a significant increase in sequence reads at the pause site compared to reads obtained at neighboring regions of the same transcript). It has been previously observed that at strong pauses such accumulations produce at least a ten fold increase in ribosome density at the pause site when compared to the full gene ribosome density [1]. We analyzed the pausing tendency of each PPX site by measuring the ratio of ribosome density between the PPX and the full gene and refer to this as the pausing index. In the wild-type strain only 14.6% of the PPX motifs had a pausing index above 2 compared to 50.4% of the PPX motifs in the Δ*efp* strain (Fig. 1A). By more stringent criteria, only 0.22% of PPX motifs had a pausing index higher than 10 in the wild-type strain compared to 2.8% in the Δ*efp* strain. Table 1 shows the 26 PPX motifs where a diprolyl or triprolyl sequence had a pausing index higher than 10 in the Δ*efp* strain. Proteomic data from SILAC showed that not all of these proteins had a significant difference in protein levels between WT and Δ*efp* strains [10]. Although both experiments were performed using different growth media, comparison of these data sets suggest that *E. coli* can compensate for decreased translation efficiency by other mechanisms related to changes in mRNA levels or protein stability. Table S1 shows the pausing index for the 16 proteins that both contain a PPX sequence and displayed at least three fold higher protein abundance in wild-type vs. the Δ*efp* strain in the *E. coli* SILAC dataset [10].

**Table 1:** EF-P dependent pauses that contain a PPX sequence.

Gene *1	PPX at pause	Upstream sequence	Pausing index *2			SILAC average E. coli WT/Δefp *3
			WT	Δ*efp*	Complemented (Δ*efp pEF-P)*	
*sgrR*	PPD	MRGLRMNTLGWFDFKSAWFA	10.75	15.77	13.79	NA
*pcnB*	PPD	ERNAELQRLVKWWGEFQVSA	4.03	21.09	11.09	1.3
*dnaE*	PPD	DVGRVLGHPYGFVDRISKLI	0.79	12.47	8.66	1.10
*ydcR*	PPG	NRSLAQVSKTATAMSVIENL	1.61	10.95	4.62	NA
*rsxC*	PPE	LKLFSAFRKNKIWDFNGGIH	3	32.97	17.87	NA
*fliI*	PPS	AMAQREIALAIGEPPATKGY	3.87	18.78	14.82	1.93
*fliP*	PPN	FTRIIIVFGLLRNALGTPSA	0	16.65	11.46	1.09
*lepA*	PPE	SAKTGVGVQDVLERLVRDIP	2.12	13.3	5.1	3.59
*lepA*	PPP	CSAKTGVGVQDVLERLVRDI	2.17	13.35	5.18	
*visC*	PPQ	SGLRVAVLEQRVQEPLAANA	2.93	12.39	8.61	2.03
*trmL*	PPN	---------MLNIVLYEPEI	0.69	11.62	5.17	NA
*rfaP*	PPD	TEDLTPTISLEDYCADWAVN	0.55	10.79	3.57	1.52
*recG*	PPP	PPELSQGMMTLPEALRTLHR	1.07	18.57	6.62	2.08
*recG*	PPT	PELSQGMMTLPEALRTLHRP	1.07	18.45	6.85	
*mnmG*	PPS	FLDGKIHIGLDNYSGGRAGD	2.72	10.57	6.5	2.54
*rffT*	PPN	LRAVHQQFGDTVKVVVPMGY	0.63	12.67	2.86	NA
*cyaA*	PPD	EQSMIEALKTILGKMHQDAA	1.21	11.78	2.89	1.17
*cyaA*	PPK	ETQRHYLNELELYRGMSVQD	3.46	11.32	7.5	
*ubiB*	PPD	AFFNRDYRKVAELHVDSGWV	0.98	20.93	8.45	1.39
*ubiD*	PPK	EDVSALREVGKLLAFLKEPE	1.05	13.88	7.62	1.53
*nfi*	PPD	RAQQIELASSVIREDRLDKD	1.35	11.24	9.51	NA
*rnr*	PPD	EAGVGFVVPDDSRLSFDILI	1.52	12.1	5.2	0.69
*ytfM*	PPP	IREGLKALGYYQPTIEFDLR	1.23	23.2	7.92	3.15
*ytfM*	PPK	REGLKALGYYQPTIEFDLRP	1.4	23.11	8.04	
*mgtA*	PPS	SRLVHRDPLPGAQQTVNTVV	6.86	16.32	12.1	0.94
*yjhB*	PPQ	----------MATAWYKQVN	10.56	45	47.34	NA

*1 : Genes with a PPPX pattern are introduced twice to account for the ribosome density at P_1_P_2_P_3_ and P_2_P_3_X.

*2 : Values correspond to averages of two independent samples.

*3 : Values from SILAC [10].

Although Δ*efp* strains showed increased ribosome occupancy at most of the PPX-encoding sequences (Fig. 1B), only a small subset of these genes had a pausing index high enough above the threshold to be considered as strong pauses (i.e. having a pausing index 10 fold above the gene average [1] (Fig. 1A). This variability holds true for all PPX patterns including many of the PPP or PPG sequences that have been reported to produce strong translation pauses in the absence of EF-P [8], [9], [11] (Fig. S2). Notable examples include *ubiD* and *malE*; both have a PPK sequence, but only translation of *ubiD* pauses at this position in the Δ*efp* strain (Fig. 1C). This indicates that other factors influence the tendency of the ribosome to pause at a particular PPX sequence. To investigate what other sequence determinants contribute to pausing we compared the strong EF-P dependent pauses (defined as regions with a pausing index of at least 10 [1] in the Δ*efp* strain) with the PPX sequences that have the lowest pausing index in the *Δefp* strain (with a pausing index equal or below 1). 31 EF-P dependent pausing sites were identified, 26 of which (distributed in 22 genes) contained a PPX motif (Table 1). The five other EF-P alleviated pauses contained no PPX motif (Table S2), consistent with our previous observation of EF-P mediated relief of non-PPX pauses such as the GSCGPG motif in the *poxB* gene [11]. These five non-PPX containing genes were further investigated by introducing the sequence coding for the pausing segment into a GFP reporter system. In this reporter GFP is in a transcriptional fusion to mCherry, which has a separate Shine-Dalgarno sequence and serves as an internal control (Fig. S3A, [11]). After inserting these non-PPX motifs into the reporter system (at the amino terminus of GFP, between codons 3 and 4), the EF-P dependency could not be reproduced (Fig. S3B). Other longer sequences were additionally tested without positive results (Table S2), suggesting that these pauses might depend at least in part on sequence features outside the cloned segments.

### Common patterns in PPX pausing motifs

The large variability in PPX-mediated pausing patterns revealed by the ribo-seq data (Fig. S2) led us to search for additional sequence features that might affect pausing at PPX sequences. We compared the sets of well-defined pausing and non-pausing motifs (Tables 1 and S3, respectively). Some patterns such as PPD or PPN were only found in the pausing PPX sequences while PPQ or PPK were present in both gene sets. When comparing alignments of the amino acid or nucleotide sequences, we were unable to identify any common patterns within either the pausing or the non-pausing PPX sequences (Fig. S4). It has been proposed that several translation pauses do not depend purely on one mechanism, but instead integrate different signals that slow down translation [4]. To investigate if other known mechanisms might contribute to pausing, the role of Shine-Dalgarno (SD) sequences upstream of the PPX sequence, the utilization of low usage tRNAs at the A site codon, and combinations of specific amino acids at the A site and upstream of PPX were tested.

### Exploring the role of the weak internal Shine-Dalgarno sequences

Most translation pauses in wild type *E. coli* are due to interactions of the mRNA's coding region with the anti-SD (aSD) sequence of the 16S rRNA [1]. We reasoned that having a motif capable of interacting with the aSD upstream of a PPX might contribute to pausing. The RNAsubopt program in the Vienna RNA package [23] was used to search for the presence of nucleotide sequences upstream of the PPX coding region that are predicted to have affinity for the aSD sequence (5′-CACCUCCU-3′), referred to here as aSD-weak binding sequences. Several paused PPX sequences also contain a sequence 7 to 9 bases upstream of the third position of the X codon from PPX predicted to weakly bind the aSD. The median affinity of these sequences for the aSD was ∼−2 Kcal/mol, about half of the minimum affinity found to produce an increased pausing index by itself in previous studies (4 to 12 Kcal/mol) [1]. It is possible that these low affinities could enhance the ability of PPX sequences to produce a pause in translation, a hypothesis that was supported by the absence of these aSD-weak binding sequences upstream of PPXs that do not produce a pause (Fig. S5A). Another possible feature of pausing patterns might be the use of rare codons that could slow translation and increase the strength of pauses [24], [25]. Consistent with this, when analyzing the codons used for PPX patterns, the stronger EF-P dependent pauses frequently use rare tRNAs for decoding the codons at the A site (Fig. S5B).

**Figure 2:**
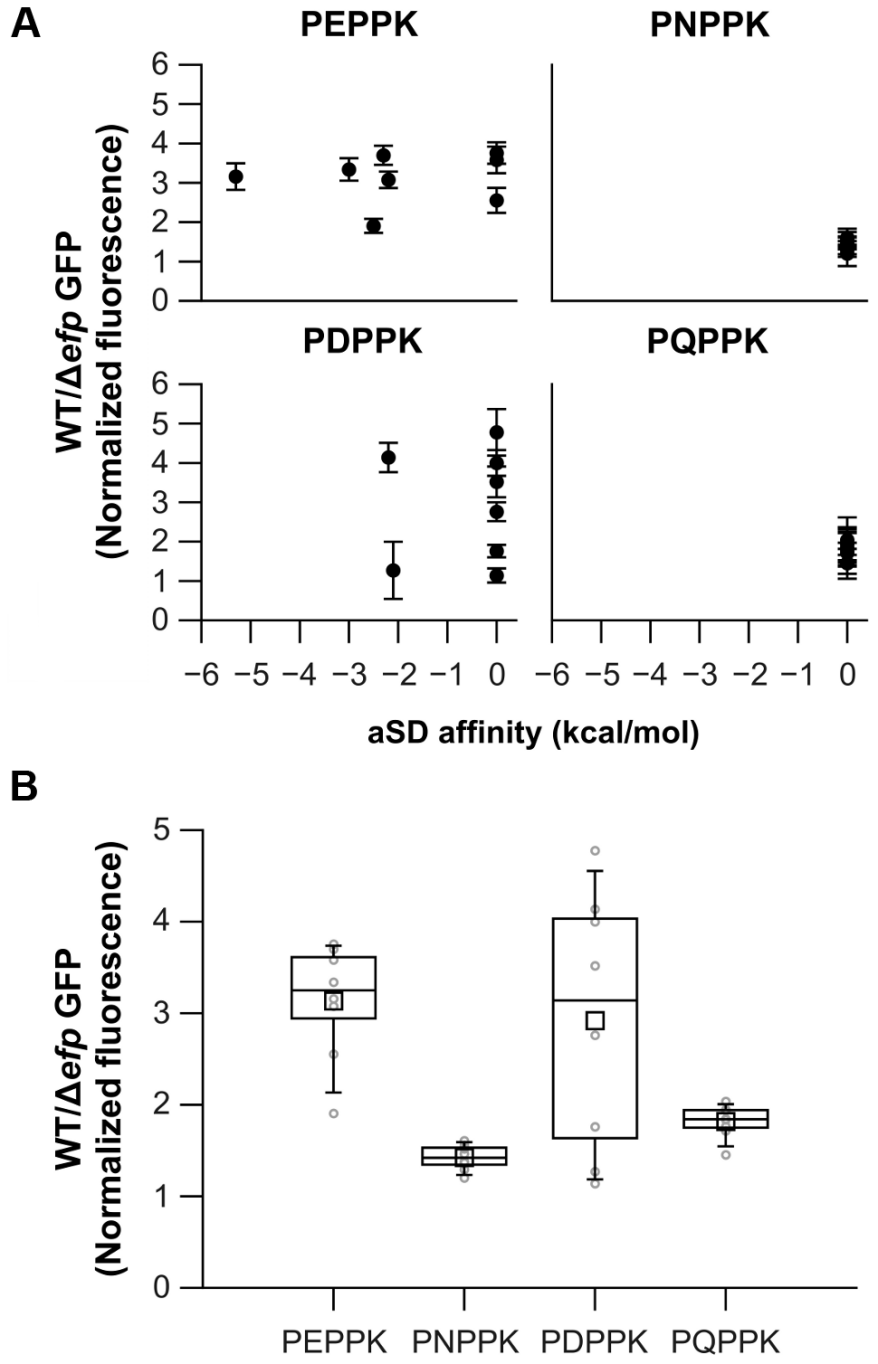
Effects on translation pausing of aSD affinity and the identity of the amino acid preceding PPX. **A**) Diverse sequences coding for similar amino acid patterns, but with varying aSD affinities, were introduced at the N-terminus of GFP. GFP production in WT and Δ*efp* strains was measured and normalized against mCherry, which is cloned as a transcription fusion. aSD affinities 6 positions upstream of the third nucleotide of the X codon are shown. **B**) Distribution of the effects of all the clones that code for the same amino acid pattern (irrespective of their specific aSD affinities) are represented as box plots in dark lines (average as a small square, mean as the middle line of the box, box limits represent 25^th^ and 75^th^ percentiles). Average values for each specific clone are shown in light gray. The mean of at least three biological replicates is shown and error bars (which indicate one standard deviation) are only shown in **A** for clarity.

**Figure 3:**
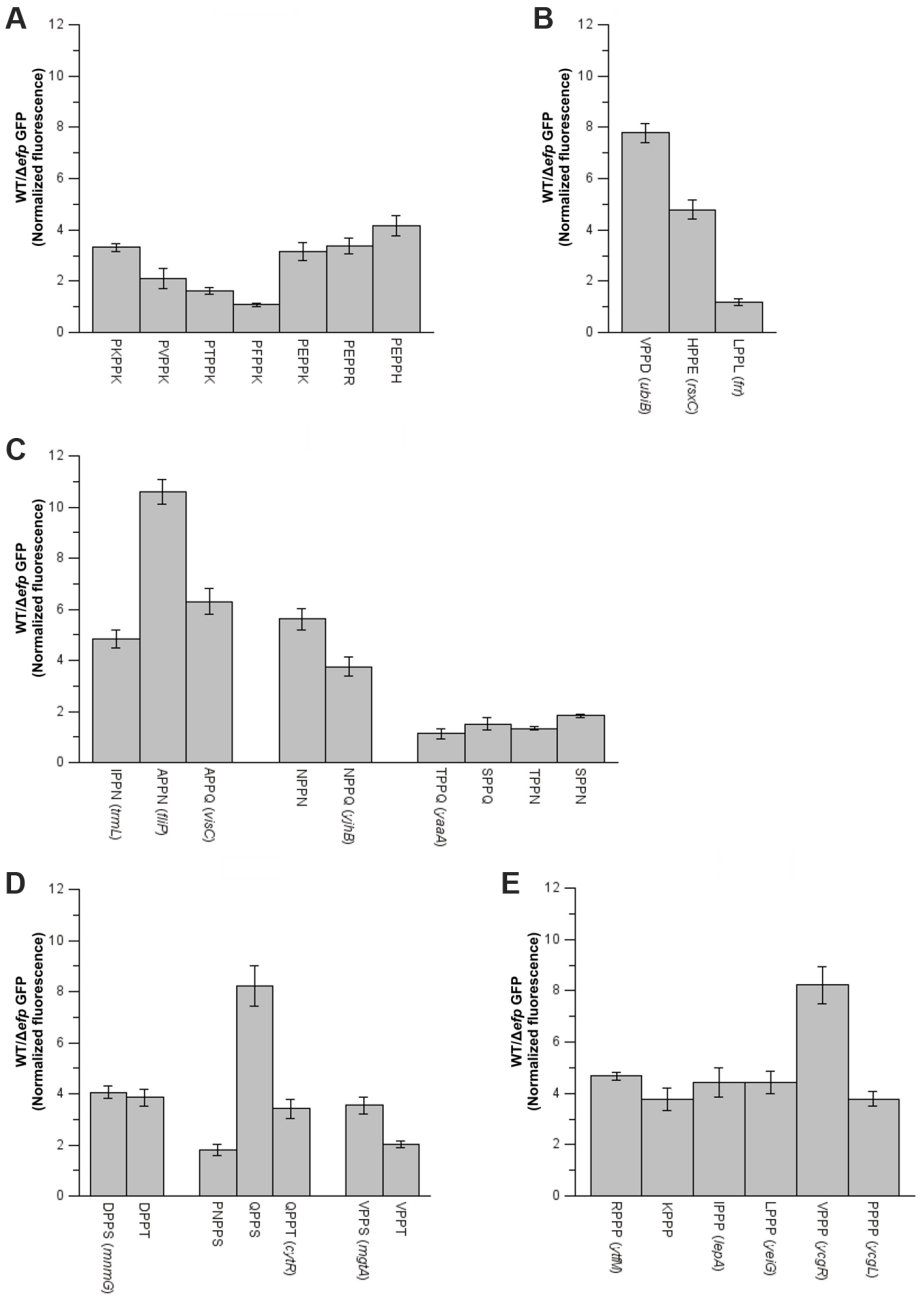
Effect of amino acids preceding PPX on pausing efficiency. Various sequences were introduced at the area coding for the N-terminus of GFP. When possible, sequences of genes found to either pause or not pause in *E. coli* were used (names in parenthesis). **A**) PP-basic, **B**) PP-acid and PP-hydrophobic, **C**) PP-amide, **D**) PP-hydroxy and **E**) PP-Pro. The mean of at least three biological replicates is shown and error bars indicate one standard deviation.

To further determine the possible role of the aSD-weak binding sequences and the use of rare codons several sequences were introduced at the amino terminus of a GFP reporter (Fig. S3A) and tested for their effect on translation in WT and Δ*efp E. coli* strains. GFP fluorescence values were normalized against the fluorescence of mCherry encoded on the same transcript immediately downstream of *gfp*. As the diverse sequence patterns are introduced at the beginning of the *gfp* sequence (between the 3^rd^ and 4^th^ codons) we do not expect that pauses have substanital effects on protein folding. Thus, the expectation is that most of the changes in GFP production associated with *efp* deletion will come from the reduction in the number of ribosomes able to cross the pausing site. This is in accordance with previous reports where comparable experiments correlated well with changes in the level of protein [10], [11]. Several codon variations coding for PEPPK were tested, a translation pause site in *ubiD*, and PNPPK found at a non-pausing segment of *malE* (Figs. 1C, 2 and Tables 1 and S3). Sequences encoding PEPPK are predicted using RNAsubopt to bind to the aSD with affinities ranging from −5 to 0 kcal/mol. Conversely, all PNPPK variants present a binding energy of 0 kcal/mol and are predicted to be easily translated by the ribosome (Fig. 2A). In addition, plasmids bearing PDPPK and PQPPK sequences were constructed as controls for the role of an acidic versus an amide containing amino acid 2 positions upstream of the P site amino acid of pausing ribosomes. GFP levels did not correlate with the sequence affinity for the ribosome aSD, indicating that this does not play a significant role in EF-P dependent pausing for P[E/D/Q/N]PPK sequences (Fig. 2A). Also, similar constructs using diverse codons at the pausing A site position did not show any effect of low usage codons (Fig. S6). Instead, a consistent tendency was observed of decreased translation efficiency for clones bearing an acidic amino acid at position −2 with regard to the Pro at the ribosome P site independent of aSD affinity (Fig. 2B). This pausing was also observed with other basic amino acids (Arg or His) at the A site position (Fig. 3A) and was independent of codon usage (Fig. S6).

### The identity of the amino acid immediately upstream of the diprolyl motif can influence the translation pausing efficiency

The finding that PP-basic pausing depends on the identity of the amino acid 2 positions upstream of the P site position (Z_−2_ on Z_−2_P_−1_P_P_X_A_), suggests a possible role of this position in determining the A site selectivity for EF-P relieved translation pausing. A similar effect has been previously observed for the macrolide dependent pausing of *ermAL1* translation, at the leader sequence of *ermA*. In this example, the presence of an Ala two positions upstream of the P-site amino acid will pause translation only in the presence of certain A site amino acids such as Glu. Conversely, the presence of Phe or Gly in the −2 position produces a non-selective ribosome that either pauses (Phe) or continues translation (Gly) irrespective of the A site amino acid [26]. Amino acids at −2 have also been shown to be important in other translation pausing examples [4] and Peil *et al.* have also recently suggested that some Z_−2_P_−1_PX patterns (with Z and X representing any proteinogenic amino acid) could also induce EF-P relieved pauses [10].

In order to determine if there is a general role of the −2 amino acid on EF-P dependent pauses, the PPX ribosome densities for all possible Z_−2_P_−1_PX amino acid combinations were analyzed (Figs. 3 and S7, Table S4). When exclusively comparing the well-defined pausing and non-pausing sequences (Tables 1 and S3, respectively) acidic amino acids at the A site (X position on Z_−2_P_−1_PX) were found to stall translation independent of the identity of the −2 amino acid (Z on Z_−2_P_−1_PX). Similarly, hydrophobic or aromatic amino acids at the A site do not produce a pause independent of the identity of the amino acid at the −2 position. Some examples of these were confirmed using the GFP/mCherry system described above (Fig. 3B). Conversely to what was observed for acidic, hydrophobic and aromatic moieties, other amino acids at the A site have a pausing behavior that is context dependent. Four examples of this variable PPX behavior were further investigated: PP-basic, PP-amide, PP-OH and PPP. Similar to previous results with PPK patterns, pausing was only observed at PP-basic motifs when the −2 residue was acidic. In contrast, PP-amides always pause with the exception of some specific cases where there is an OH containing amino acid at the −2 position. These activities were confirmed for both patterns in the GFP/mCherry system, although acidic-PP-basic patterns have a weak effect on GFP translation as compared to the other patterns analyzed (Figs. 3A and 3C).

In ribosome profiling, PP-OH was only observed to pause when the A site was occupied by a Ser, whereas the presence of Tyr or Thr did not cause a translation pause. In the GFP/mCherry reporter system, PP-S and PP-T produced some decrease of translation in the Δ*efp* strain. In some cases (with Gln or Val preceding PPX) Ser in the A site produced a stronger effect than Thr, but in others (with Asp preceding PPX) no difference was observed (Fig. 3D). Contrary to predictions based on previous reports, PPP sequences were only observed to pause with an Arg or Ile at −2 in the ribosome profiling data. The effect of amino acids at the −2 position was also studied using the GFP system (Fig. 3E). All PPP motifs produced at least a 4-fold decrease in GFP production in the Δ*efp* strain compared to WT. Previous studies have suggested that longer Pro stretches will induce stronger pauses. For instance, we have previously shown that a 6 Pro stretch will reduce GFP translation 3- to 4-fold more than a 3 Pro stretch [11]. By contrast, the addition of only one Pro before PPP (PPPP) does not have any effect on either GFP expression or ribosome occupancies in ribosome profiling experiments (Figs. 3E and S7, Table S4) indicating that addition of a single prolyl residue is not enough to significantly reduce translation efficiency.

### Effects of distal upstream sequences on PPX translation

The finding that all ZPPP motifs produced a ∼4 fold effect in the GFP/mCherry reporter system was unexpected, as in the ribo-seq data only RPPP and IPPP were observed to produce a strong pause. Moreover Val, that only appeared preceding non-pausing PPP in our ribo-seq data, had the strongest effect compared to the other ZPPP patterns (Figs. 3E and S7). The finding that some of the patterns tested in the reporter system were unable to reproduce the pausing tendency observed in ribo-seq suggests that other sequence features might have additional effects on the pausing of PPX. No obvious correlation was observed between the “X” amino acid and up to 12 codon positions further upstream of PPX in our set of validated *in vivo* pausing sequences.

**Figure 4:**
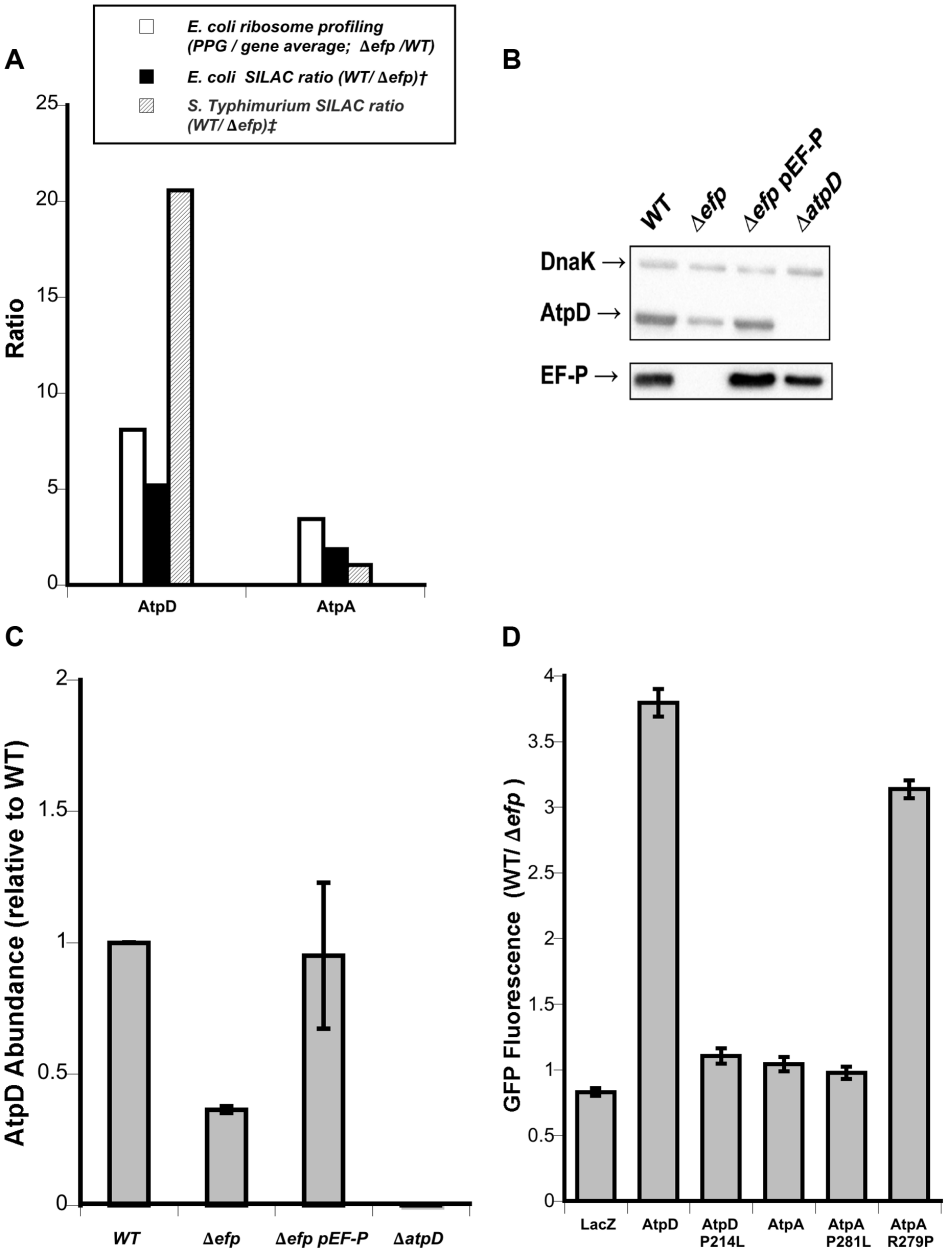
Dependence on EF-P of AtpA and AtpD synthesis. **A**) Ribosome profiling and SILAC ratios comparing *atpD and atpA* translational stalling and AtpD and AtpA protein levels in *E. coli* and *Salmonella* Typhimurium. Ribosome profiling is from this work; † *E. coli* SILAC data from [10]; ‡ *S.* Typhimurium SILAC data from [11]. **B**) Representative western blot showing AtpD levels in strains of *Salmonella*. DnaK was included as a loading control. **C**) Densitometry quantification of western blots showing mean AtpD/DnaK ratio relative to WT across three biological replicates. Error bars indicate one standard deviation. **D**) Fluorescence ratios comparing synthesis of plasmid-encoded AtpD- and AtpA-GFP translational fusions in wild-type (WT) and *efp* mutant *Salmonella*. LacZ is included as a control and specific point mutations affecting the PPG motifs are indicated. Ratios show WT/Δ*efp* for GFP fluorescence at 10 hours post-inoculation normalized to optical density (600 nm). The mean of at least three biological replicates is shown and error bars indicate one standard deviation.

To more broadly explore the contextual effect of larger sequences on EF-P dependent translation the expression efficiency of the *atpA* and *atpD* genes was compared in *Salmonella*. Both genes encode for similar proteins that contain a PPG motif and are expressed from the same mRNA transcript. However, proteomic analysis of *Salmonella* showed that only *atpD* expression appeared to be affected by *efp* deletion (20.6 fold difference expression for *atpD* contrasting with 1.05 for *atpA*) [11]. A similar trend, although less dramatic, was recently observed in a SILAC experiment performed with *E. coli* (5.18 fold difference in synthesis for *atpD* and 1.88 for *atpA*) [10]. Conversely, in the current ribosome profiling experiment, the pausing index at PPG in the Δ*efp* strain is similar between the genes, 3.80 for *atpA* and 5.04 for *atpD* (Fig. 4A).

To verify the results from the high-throughput analyses, we conducted western blotting that confirmed the AtpD protein level is lower in an *efp* mutant of *Salmonella* (Fig. 4B, quantified in 4C). We then addressed the discrepancy in EF-P dependent expression of *atpA* and *atpD* by employing the previously used pXG10sf translational fusion system to compare translation in wild type and *efp* mutant *Salmonella* [11], [27], [28]. The constructs allowed for the constitutive transcription of mRNA bearing full-length *atpA* or *atpD* genes with “super-folder” GFP as a C-terminal translational fusion [29]. Consistent with proteomic analysis, the fluorescence measurements revealed that the expression of *atpD* was dependent on EF-P whereas *atpA* was not (Fig. 4D). Mutation of the PPG motif to PLG (P214L) abolished the EF-P requirement for *atpD* and, conversely, lengthening the PPG of *atpA* to PPPG (R279P) induced strong EF-P dependence. Unsurprisingly, mutation of the *atpA* PPG motif to PLG (P281L) did not have a significant affect as *atpA* expression was already independent of EF-P. No change in EF-P dependence was observed for either construct upon switching the second proline codon or by altering the upstream codons to strengthen or weaken binding to the aSD sequence of 16s rRNA (Fig. S8), consistent with results described above.

**Figure 5:**
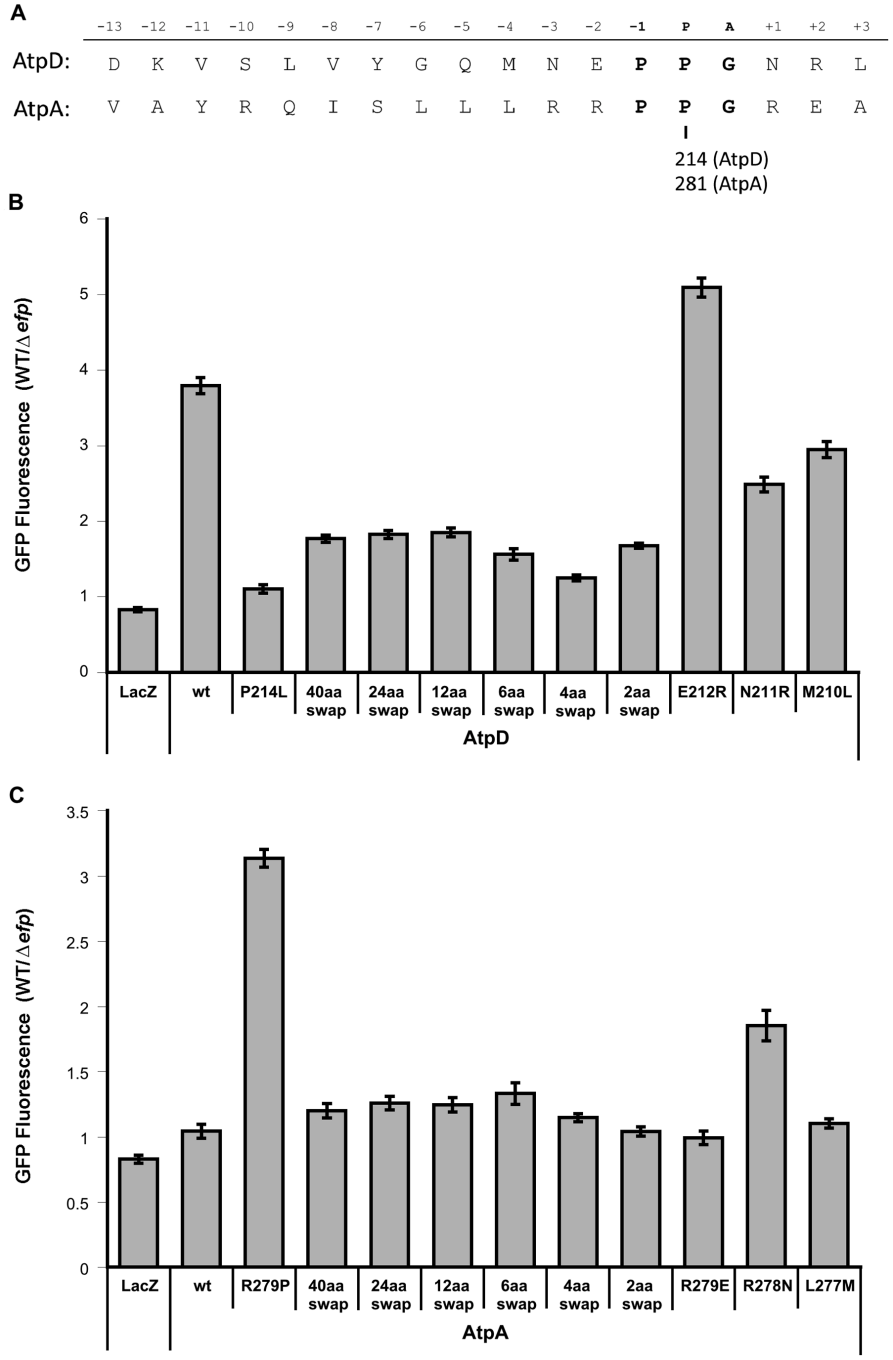
Effect of upstream residues on the EF-P dependence of AtpD and AtpA synthesis. **A**) Sequences (*Salmonella* Typhimurium) of AtpD and AtpA in proximity to their PPG motifs (bold). The relative position when the glycine of PPG occupies the A site is shown above. The amino acid position of the second proline of the PPG motif in each protein is indicated below. **B**) Fluorescence ratios comparing expression of plasmid-borne AtpD-GFP translational fusions in wild-type (WT) and *efp* mutant *Salmonella*. ‘Swap’ constructs indicate swap-in of AtpA sequence for the specified number of amino acids upstream of the PPG motif. LacZ, unmodified (wt), P214L and R279P constructs from Figure 4 are included for comparison. Ratios show WT/Δ*efp* for GFP fluorescence at 10 hours post-inoculation normalized to optical density (600 nm). The mean of at least three biological replicates is shown and error bars indicate one standard deviation. **C**) As in **B**, but with AtpA-GFP translational fusion constructs with swap-in of AtpD upstream sequence.

Since interactions between the nascent polypeptide chain and the ribosomal exit tunnel can affect translational stalling, the role in EF-P dependence of the regions upstream of the *atpD* and *atpA* PPG motifs was also investigated [2], [4], [30]–[32]. Up to 40 codons upstream of the *atpD* PPG motif were swapped into the pXG10sf-*atpA* construct while leaving the PPG motif and the remainder of the ORF intact, or vice versa. Swapping as few as two upstream codons from *atpA* into *atpD* led to a significant increase in expression of the *atpD-gfp* construct in the *efp* mutant of *Salmonella* (Figs. 5 and S9). This effect increased when four codons were swapped, but returned to a similar degree when six or more residues were switched. A similar reversal of EF-P dependence was observed for swapping upstream regions of *atpD* into *atpA*: a four codon swap led to a very small increase in EF-P dependence, which increased only marginally when six or more codons were swapped. The observation that swapping greater than six residues upstream had marginal or no additional effect suggests that, at least in this instance, the important interactions with the ribosomal exit tunnel are occurring close to the peptidyl transferase center and prior to the exit tunnel constriction that has been implicated in other extended translational stall motifs [2], [31], [33].

Interestingly, no effect was observed for either *atpA* or *atpD* when the Z_−2_ amino acid was swapped (E212R for AtpD; R279E for AtpA). However, swapping of the Z_−3_ position residues resulted in a drastic effect on EF-P dependence (N211R for AtpD; R278N for AtpA) (Fig. 5). The *atpD* N211R mutant construct partially alleviated EF-P dependence, though to a lesser degree than the Z_−3_Z_−2_ amino acid swap. In contrast, changing the Z_−3_ position of *atpA* to arginine as found in *atpD* (R279N) led to a dramatic increase in EF-P dependence, surpassing the effect of all other constructs where more residues were altered. Thus, while a common pattern could not be detected within the pausing PPX genes, at least in the case of *atpA/D* (and possibly of other PPG containing genes) the residue three positions upstream of the P site plays an important role in determining whether progression through PPG motifs will depend on EF-P. Furthermore, the data shows that other nearby residues can dampen this effect.

## Discussion

### Ribosome profiling and protein synthesis

A challenging aspect of analyzing ribosome profiling data is that increased density of ribosome footprints can indicate many ribosomes actively translating a transcript or an increased translation time [20]. The ribosome profiling data introduced here was compared with the available proteomic data obtained by SILAC [10] of wild type *E. coli* and Δ*efp* strains. Upon comparing total footprints/gene ratios for Δ*efp*/WT strains (obtained by ribo-seq) to differences in protein abundance for Δ*efp*/WT strains detected by SILAC, most proteins (77%) seem unaffected, with only 2% of proteins showing a greater than 2-fold increase in both datasets (Fig. S10). This comparison also showed that 5.5% of the proteins having 2-fold higher protein abundance as detected by SILAC show decreased or unchanged ribosome occupancies from ribo-seq. This could be a result of many factors including differences in protein half-life or mRNA abundance between WT and Δ*efp* strains. In this sense it is particularly interesting to note that synthesis of RNase II, which plays a critical role in mRNA turnover, is highly EF-P dependent. Ribo-seq showed that the PPQ motif in *rnb* (encoding RNAse II) had a 7.4 pausing index (Table S1) while SILAC detected RNase II to be 7 times more abundant in the WT versus the Δ*efp* strain [10].

The differences between Ribo-Seq and SILAC may also be influenced by inherent biases of the ribosomal profiling method [34]. The protocol involved in generating footprints captures short mRNA fragments covered by exactly one ribosome. It remains possible that mRNA fragments with very closely located ribosomes (as we expect near pausing sites) could be lost as has been previously shown for other pauses [35]. Moreover, determining pausing sites by computing motif reads divided by gene average [1] can be misleading when the gene has more than one pausing motif or when the pausing motif is at the start of the gene; in both of these cases the average reads/gene would probably be inaccurate.

### Defining sequence elements that require EF-P for efficient translation

The results presented here confirm previous observations that most PPX motifs do not require EF-P for proper translation [10], [11]. Instead, potential EF-P alleviated pauses are restricted to a small subset of these proteins. With the exception of PPG and PPP that have special structural features, all the strong EF-P dependent pauses found in the ribosome profiling data (Table 1) have a polar amino acid at the A site. Trp at the A site has been previously found to produce translation pausing *in vitro* [4], [10], but could not be detected in our *in vivo* ribosome profiling data. Similar results have been previously obtained through proteomic data [10]. This suggests a possible effect of some polar groups on the positioning of the amino acid moiety of aminoacyl-tRNA at the A site of ribosomes. More importantly, our data show a dependency of PPX pauses on the identity of amino acids located N-terminal of the pausing site. This is in accordance with recent reports from Peil *et al.* indicating that amino acids like Asp, Ala or Ile can stimulate EF-P dependent pauses if located just upstream of a PPX sequence [10]. In contrast to their report, our results indicate that the effects of preceding amino acids are highly dependent on the context in which they are located. For instance, an Asn will prevent pausing when located before PPK, but allow it when located next to PP [N/Q] (Fig. 3). More striking is a comparison of the pausing strength of [Q/V]PPS to [Q/V]PPT or of QPPS to NPPS (Fig. 3D). In both cases a single methyl group is enough to determine whether or not translation pauses. This shows an exquisite level of selectivity at either the PTC or the exit tunnel, both of which are usually expected to be fairly non selective in order to facilitate synthesis of all proteins.

Our finding that amino acids located two residues upstream of PPX (the Z_−2_ and Z_−3_ positions) influence pausing is supported by our previous finding that the motif generating the EF-P alleviated pause in PoxB requires a stretch of 6 amino acids [11]. Moreover, in this work we now observe that the region upstream of the PPG motifs in *atpA* and *atpD* can significantly influence EF-P dependence, with particular emphasis on the −3 position. Other residues located further upstream can also modulate the strength of the PPX pause (Fig. S9). One possible explanation for these results is that interactions between the nascent peptide and the exit tunnel modulate the selectivity of the A site, similar to what has been observed for the macrolide relieved pausing of *ermAL1* translation [26] or the translocon relieved translation of *secM* [36]. In these two cases the role of the amino acid two positions upstream of the P site is as important as that observed here for most EF-P relieved pauses. In contrast, pausing during translation of TnaC seems to depend on the −3 position [37], similar to what we observed for *atpD* translation. Effects from positions further upstream also have relevant roles for some EF-P relieved pauses, as we have previously observed for the non-PPX pause on PoxB translation that depends on 6 continuous amino acids [11]. Thus, EF-P relived pauses depend on an array of diverse amino acid sequence contexts that interact with the PTC or the exit channel. Additional local effects of mRNA structure or interactions with the ribosome can not be ruled out as we did observe some variability in GFP expression depending on codon usage in the regions upstream of PPK (Fig. 2A). Nevertheless, these effects were usually small and did not correlate with either codon usage or affinity for the ribosomal aSD.

### The role of EF-P in integrating different signals to regulate translation

We were surprised that only 2.8% of the PPX motifs detected by ribosome profiling had a pausing index of 10 or more (the threshold considered as strong pausing) [1]. Although, the remainder of the PPX motifs may not be pausing translation, the observed increase in ribosome density likely reflects that many PPX motifs can still slow translation. This might offer some explanation for the increased polysome retention previously observed for *E. coli* Δ*efp* strains [17]. Similar effects are also observed after addition of chloramphenicol to *E. coli* cultures [17] or depletion of eIF5A (EF-P paralog) in eukaryotic cells [38]–[40]. These observations suggest that EF-P enhances the translation speed of several slightly slower segments of mRNA that collectively would have an important effect on global translation dynamics. Part of this translation enhancement may not come directly from EF-P binding to the ribosome, but from the release of tRNA^Pro^ that is trapped in other stalled ribosomes.

Both ribosome profiling and SILAC data show that EF-P directly affects the synthesis of several key components of translation, and therefore the loss of EF-P may have a broad but indirect impact on protein synthesis. YjjK (or EttA, Energy-dependent translational throttle A [41]) has recently been shown to be sensitive to the ATP/ADP ratio in the cell. EttA can control the progression of 70S ribosome initiation complexes into translation elongation and thus alter protein synthesis in energy-depleted cells [41], [42]. EttA has 2 PPX motifs, PPG and PPK, and in the ribo-seq data PPG caused a pausing index of 3.1 in the Δ*efp* strain, a ∼14 fold increase compared to WT (Table S2). Similarly, SILAC data showed that Etta is 3.5 and 15.4 times more abundant in WT than in *efp* deletion strains in *E. coli* and *Salmonella*, respectively [10], [11]. In view of the fact that components of ATP synthase and also Etta are affected by the loss of EF-P [10], [11], it is conceivable that loss of EF-P may perturb the energy state of the cell, which may in turn contribute to the growth defect of the Δ*efp* mutant. Our ribo-seq data also shows that another translation factor, LepA, has 2 PPXs that both caused a pausing index higher than 13.3, and SILAC data showed a significant change in the WT/Δ*efp* ratio of 3.6. (Table 1 and S1 [10]). Changes in the levels of other proteins observed in the *E. coli* SILAC data [10] could further affect protein translation. Examples of this are RaiA (a translation inhibitor and ribosomal stability enhancer [43] that shows 8 fold increase in Δ*efp* strain), Sra (a protein of unknown function that binds 30S ribosomal subunits during stationary phase [44] and shows a ∼2 fold increase in Δ*efp* strain) and several proteins involved in tRNA processing (RNaseII(Rnb) [45]), modification (MnmE, MnmG, SelU [46], [47]) or aminoacylation (LysU, ValS [48]) that present 2- to 7-fold reduced levels in the Δ*efp* strain. In addition, SILAC data shows a ∼2 fold decrease in the levels of the chaperone HslU in the Δ*efp* strain. As this chaperone is also part of the HslVU protease [49], changes in its levels could affect protein stability. Additionally, a 3 fold increase in the levels of HchA(Hsp31) in the Δ*efp* strain could also produce changes in protein stability as this chaperone has been proposed to have some proteolytic activity [50].

Altogether, changes in the levels of proteins involved in protein synthesis and stability could explain some of the differences we observe between ribo-seq and SILAC data. Additionally, the effects observed here on cellular levels of translation factors such as EttA and LepA, on proteins modifying protein stability such as HslU and on proteins expected to influence mRNA turnover such as RNaseII, suggest a broad role for EF-P in integrating and balancing different inputs that determine the efficiency of protein synthesis.

## Materials and Methods

### General methods

*E. coli* BW25113 (Wild type) and Δ*efp E. coli* strains were obtained from the Keio collection. For the Δ*efp* strain, the kanamycin cassettes was removed via pCP20-encoded FLP recombinase and was confirmed by PCR [51], [52]. The Δ*efp E. coli* complemented strain was constructed by introducing the *efp* open reading frame in *trans* on the arabinose-inducible vector pBAD (Δ*efp* pEF-P strain). Plasmids used for motif verification were derivatives of pBAD30 [53]. As previously described [11], the plasmid contained a tandem fluorescent fusion cassette composed of green fluorescent protein (*gfp*) followed directly by *mCherry*. A cloning site was added to that construct after the 3^rd^ codon of *gfp*. This plasmid was subsequently designated pBAD30XS. Patterns were inserted into pBAD30XS by double strand oligo hybridization.

Translational fusion experiments assessing the expression of *atpA* and *atpD* were conducted in *Salmonella enterica* serovar Typhimurium strain 14028s (referred to as *Salmonella* in body text) and an isogenic *efp* deletion mutant designed to avoid interference with the *yjeK* promoter [11]. The open reading frame of *atpA* or *atpD*, plus 75 or 74 bp upstream (respectively) were inserted into the NheI and NsiI sites of the pXG10sf plasmid employed previously [11], [27], [28]. The plasmid employs a tightly controlled, low-copy number origin of replication (pSC101) and the constitutively active PLtet0-1 promoter to minimize variation in transcript levels. Mutations and swaps were generated using site-directed mutagenesis or Gibson isothermal assembly cloning using overlapping primers [54]. All strains, plasmids and primers used in this study are described in Table S6.

### Ribosome profiling

Saturated cultures of Wild type *E. coli* BW25113, Δ*efp* and the complemented strain were diluted to an OD_600 nm_ of 0.01 in 200 mL of Luria broth medium. The media for the Δ*efp* complemented strain was supplemented with 0.02% arabinose. Strains were grown at 37°C, 250 rpm to OD_600 nm_ 0.4–0.5. Cells were harvested by rapid filtration [22] through a prewarmed 0.45 µm nitrocellulose membrane; the cells were scraped onto a pre-warmed spatula then directly submerged in liquid nitrogen. The frozen cells were dislodged into 0.65 ml of cold lysis buffer [20 mM Tris-HCl pH 8.0, 10.5 mM MgCl2, 40 U/µl RNase Inhibitor (Roche), and 100 U/ml Turbo DNase (NEB)] [17] and re-chilled in liquid nitrogen. The harvested cells were lysed by freeze/thaw 3 times then spun down at full speed for 10 min at 4°C. The clarified supernatant was immediately frozen in liquid nitrogen and stored at 80°C. This lysate was used to prepare footprint and total mRNA samples. A detailed description of sample preparation and library generation is provided in Text SI.

### Data analysis

Ribosome profiling sequence reads were trimmed and aligned to the *E. coli* K12 MG1655 reference genome (Genebank version U00096.2) using FASTX-Toolkit and bwa (0.6.2) [55]. A file describing the coverage for each feature in the *E. coli* genome, was created using bedtools (2.17.0). Reads in each gene were normalized by the average of reads in the whole ORF and pauses were identified searching for peaks where ribosome occupancies were at least 10 fold above the gene average. A detailed description of these and other sequence analyses is provided in Text SI. Data has been deposited in the NCBI Sequence Read Archive (SRA) BioProject no. PRJNA241328.

### Pausing-pattern verification

Overnight cultures of *E. coli* strains harboring pBAD30XS constructs in LB were diluted to an optical density at 600 nm (OD_600_) of 0.05 in M9 minimal salts medium supplemented with 0.4% (wt/vol) glycerol, 100 µg/ml ampicillin and 0.2% arabinose. All cultures were incubated at 37°C. Fluorescence was assessed using a spectrofluorimeter (Horiba) after 10 hrs. Cells were analyzed for GFP using excitation at 481 nm and emission at 507 nm and for mCherry with excitation at 587 nm and emission at 610 nm. The background level with blank medium was subtracted, and the ratio of GFP fluorescence over that of mCherry was calculated. Reported values represent averages and standard deviations determined from three independent experimental replicates.

### GFP fluorescence assay for translational fusions of atpA and atpD

As described previously, LB overnight cultures of wild type or *efp* mutant *Salmonella* bearing a *atpD* or *atpA* constructs in pXG10sf were diluted 1/200 into MOPS minimal medium supplemented with 0.2% glucose and 20 µg/ml chloramphenicol [11]. The cultures were grown for 16 hrs at 37°C with shaking in a Tecan Infinite M200 microplate reader. Fluorescence (475 and 511 nm excitation and emission wavelengths, respectively) and OD_600_ were measured every 15 min. Background from media-only controls was subtracted and data was expressed as GFP fluorescence per OD_600_ unit at 10 hr post-inoculation.

### Immunoblotting and quantification

*Salmonella* strains were grown in MOPS minimal media supplemented with 0.2% glucose to mid log phase (OD_600_∼0.5), washed twice (1 mM Tris pH 8.0, 5 mM magnesium acetate) and lysed by sonication in lysis buffer (9.32 M urea, 2.67 M thiourea, 40 mM Tris, 86.78 mM CHAPS, pH 8.5). 10 µg of total cell lysate was mixed with 2× SDS loading buffer and boiled for 10 min at 95°C. Proteins were separated by SDS-PAGE and transferred (semidry) to a nitrocellulose membrane. Following 1 h blocking at room temperature in 5% milk in TBST (1× Tris-buffered saline, 0.05% Tween 20), immunoblotting was conducted overnight in TBST +5% mile at 4°C using a mouse anti-DnaK antibody (1∶50,000; Enzo Life Sciences) and a mouse monoclonal antibody specific for the beta subunit of *E. coli* ATP Synthase (1∶1000; MitoSciences). Blots were washed and subsequently incubated for 1 h at room temperature with HRP-fused goat anti-mouse antibody (1∶10,000 in TBST +5% milk) for ECL imaging (Thermo Scientific). Quantification of AtpD protein levels relative to DnaK was done using *Image Lab* software (Bio-Rad Laboratories).

## Supporting Information

Figure S1 Correlation for biological replicates of ribosome profiling data from Wt, Δ*efp* and the complemented strains (Δ*efp* pEF-P). Our experiment resulted in an average of 12.2 million reads/sample. Scatter plots show correlation of total footprints/gene (log10) for the biological replicates.
(TIF)

Figure S2 Comparison of pausing index for each PPX. Box plot comparing the PPX pausing indices of every possible PPX combination. Pausing index was calculated for all PPX with significant amount of reads in the ribo-seq. Distribution of all the pausing index found for each PPX are shown in a box plot (average as a small square, median as the middle line of the box, box limits represent 25^th^ and 75^th^ percentiles, higher and lower values are marked with an “x” symbol).
(TIFF)

Figure S3 The GFP/mCherry reporter. A) Schematic of the GFP/mCherry reporter: GFP is in a transcriptional fusion to mCherry that has a separate Shine-Dalgarno sequence. mCherry serves as an internal control for variations in transcription and plasmid copy number [11]. Tested motifs were inserted in-frame at the fourth codon of *gfp* [56]. Fluorescence ratio (GFP/mCherry) is measured for WT and Δ*efp* strains harboring the tested plasmid. The fold difference in fluorescence ratios between WT and Δ*efp* strains is then normalized to the values obtained from a no insert control. B) The reporter construct with the 5 non-PPX motifs described in Table S2. The five motifs have fluorescence ratios lower than no insert, PPPPPP is the positive control.
(TIF)

Figure S4 Alignment of sequences from genes with pausing or non-pausing PPX. Figure shows a Logo representation [57] of alignments of nucleotide (**A** and **B**) or amino acid (**C** and **D**) sequences of pausing (**A** and **C**) or non pausing (**B** and **D**) PPX containing genes. PPX sequences were manually aligned together with the first 20 upstream codons/amino acids. No gaps were introduced in the sequence.
(TIF)

Figure S5 Correlations between mRNA sequence and pause strength. **A**) Figure shows estimated affinity for ribosomal aSD sequence in sequences upstream of PPX in pausing and non-pausing genes. Position o correspond to the third position of the X codon at PPX (average as a small square, median as the middle line of the box, box limits represent 25^th^ and 75^th^ percentiles, higher and lower values are marked with an “x” symbol). **B**) Comparison of codon usage for all PPX sequences and their corresponding ribosome occupancies in the strain. Values correspond to average of two biological replicates and are normalized by the average occupancies of the corresponding genes.
(TIF)

Figure S6 Effect of codon usage at A site position.
(TIF)

Figure S7 PPX to gene occupancy ratios for all ZPPX combinations found on *E. coli*. PPX to gene occupancy ratios for all possible amino acid combinations between the last amino of PPX and the one that is immediately upstream of it was plotted. One graph was made for each PPX.
(TIF)

Figure S8 Altering anti-Shine Dalgarno sequence binding or Pro214 codon does not affect expression of AtpD or AtpA. Fluorescence ratios comparing expression of codon mutations in AtpD- and AtpA-GFP fusion constructs in pXG10sf maintaining amino acid sequence while altering binding to the anti-Shine Dalgarno sequence of 16s rRNA, or altering the second proline codon of the AtpD PPG motif (CCG) to the CCA pro codon in that position in AtpA. LacZ and unmodified (wt) AtpD and AtpA constructs from ***Figure 4*** are included for comparison. Ratios show WT/Δ*efp* (*Salmonella Typhimurium*) for GFP fluorescence at 10 hours post-inoculation normalized to optical density (600 nm). The mean of at least three biological replicates is shown and error bars indicate one standard deviation.
(TIF)

Figure S9 Extended mutagenesis of residues upstream of AtpD and AtpA PPG motif. **A**. As in ***Figure 5***, showing sequence (*Salmonella Typhimurium*) of AtpD and AtpA in proximity to their PPG motifs (bold). The relative position when the PPG glycine occupies the A site is shown above. The amino acid position of the second proline of the PPG motif in each protein is indicated below. **B**. Constructs generated in addition to those shown in ***Figure 5***. Values are fluorescence ratios comparing expression of plasmid-borne AtpD-GFP translational fusions in wild-type (WT) and *efp* mutant *Salmonella*. Unmodified (wt) and 12aa swap construct from ***Figure 5*** are shown for comparison. Ratios show WT/Δ*efp* for GFP fluorescence at 10 hours post-inoculation normalized to optical density (600 nm). The mean of at least three biological replicates is shown and error bars indicate one standard deviation. **C**. As in **B**, but with AtpA-GFP translational fusion constructs with swap-in of AtpD upstream sequence.
(TIF)

Figure S10 Comparison of SILAC and rib-seq data. Pie chart comparing proteins identified in our ribo-seq experiment (cutoff 70 footprint reads/gene) and also present in Peil *et al.*, SILAC dataset [10]. Ribo-seq data (ratio between Δ*efp* and WT footprints/gene) was compared with SILAC data (protein abundance ratio between Δ*efp* and WT). In 77% (800 out of 1039) of the proteins, the ratio for both datasets was between 0.5–2. For 7.5% of the genes, there was more than 2 fold higher total footprints/gene in Δ*efp* vs. WT, about one fourth of them also had above 2 folds more protein abundance in Δ*efp* vs. WT. While 7.3% of the genes had less had less than 0.5 fold footprints/gene in Δ*efp* vs. WT, about one tenth of them also had less than 0.5 fold protein abundance in Δ*efp* vs. WT.
(TIF)

Table S1 List of PPX-containing proteins that were identified by SILAC [10] to be 3-fold or more abundant in the wild-type vs. Δ*efp* strain and their corresponding pausing index from ribo-seq.
(DOC)

Table S2 List of EF-P dependent pauses that do not contain a PPX sequence.
(DOC)

Table S3 List of PPX sequences that do not produce a translation pause.
(DOC)

Table S4 Combinations of amino acids at A site (X, #10) and 2 positions upstream the P site (Z) in pausing and non-pausing ZPPX sequences.
(DOC)

Table S5 Indexed library PCR primers.
(DOCX)

Table S6 Strains, plasmids and primers used in this study.
(DOC)

Text S1 Additional experimental procedures.
(DOCX)

## References

[1] Li GW, Oh E, Weissman JS (2012) The anti-Shine-Dalgarno sequence drives translational pausing and codon choice in bacteria. Nature 484: 538–541. 22456704 10.1038/nature10965 PMC3338875

[2] Ito K, Chiba S, Pogliano K (2010) Divergent stalling sequences sense and control cellular physiology. Biochem Biophys Res Commun 393: 1–5. 20117091 10.1016/j.bbrc.2010.01.073 PMC2885147

[3] Lu J, Deutsch C (2008) Electrostatics in the ribosomal tunnel modulate chain elongation rates. J Mol Biol 384: 73–86. 18822297 10.1016/j.jmb.2008.08.089 PMC2655213

[4] Woolstenhulme CJ, Parajuli S, Healey DW, Valverde DP, Petersen EN, et al (2013) Nascent peptides that block protein synthesis in bacteria. Proc Natl Acad Sci U S A 110: E878–887. 23431150 10.1073/pnas.1219536110 PMC3593848

[5] Yap MN, Bernstein HD (2009) The plasticity of a translation arrest motif yields insights into nascent polypeptide recognition inside the ribosome tunnel. Mol Cell 34: 201–211. 19394297 10.1016/j.molcel.2009.04.002 PMC2704006

[6] Komar AA, Lesnik T, Reiss C (1999) Synonymous codon substitutions affect ribosome traffic and protein folding during in vitro translation. FEBS Lett 462: 387–391. 10622731 10.1016/s0014-5793(99)01566-5

[7] Tsai CJ, Sauna ZE, Kimchi-Sarfaty C, Ambudkar SV, Gottesman MM, et al (2008) Synonymous mutations and ribosome stalling can lead to altered folding pathways and distinct minima. J Mol Biol 383: 281–291. 18722384 10.1016/j.jmb.2008.08.012 PMC2628389

[8] Doerfel LK, Wohlgemuth I, Kothe C, Peske F, Urlaub H, et al (2013) EF-P is essential for rapid synthesis of proteins containing consecutive proline residues. Science 339: 85–88. 23239624 10.1126/science.1229017

[9] Ude S, Lassak J, Starosta AL, Kraxenberger T, Wilson DN, et al (2013) Translation elongation factor EF-P alleviates ribosome stalling at polyproline stretches. Science 339: 82–85. 23239623 10.1126/science.1228985

[10] Peil L, Starosta AL, Lassak J, Atkinson GC, Virumae K, et al (2013) Distinct XPPX sequence motifs induce ribosome stalling, which is rescued by the translation elongation factor EF-P. Proc Natl Acad Sci U S A 110: 15265–15270. 24003132 10.1073/pnas.1310642110 PMC3780873

[11] Hersch SJ, Wang M, Zou SB, Moon KM, Foster LJ, et al (2013) Divergent protein motifs direct elongation factor P-mediated translational regulation in Salmonella enterica and Escherichia coli. MBio 4: e00180–00113. 23611909 10.1128/mBio.00180-13 PMC3638311

[12] Hanawa-Suetsugu K, Sekine S, Sakai H, Hori-Takemoto C, Terada T, et al (2004) Crystal structure of elongation factor P from Thermus thermophilus HB8. Proc Natl Acad Sci U S A 101: 9595–9600. 15210970 10.1073/pnas.0308667101 PMC470720

[13] Yanagisawa T, Sumida T, Ishii R, Takemoto C, Yokoyama S (2010) A paralog of lysyl-tRNA synthetase aminoacylates a conserved lysine residue in translation elongation factor P. Nat Struct Mol Biol 17: 1136–1143. 20729861 10.1038/nsmb.1889

[14] Blaha G, Stanley RE, Steitz TA (2009) Formation of the first peptide bond: the structure of EF-P bound to the 70S ribosome. Science 325: 966–970. 19696344 10.1126/science.1175800 PMC3296453

[15] Roy H, Zou SB, Bullwinkle TJ, Wolfe BS, Gilreath MS, et al (2011) The tRNA synthetase paralog PoxA modifies elongation factor-P with (R)-beta-lysine. Nat Chem Biol 7: 667–669. 21841797 10.1038/nchembio.632 PMC3177975

[16] Peil L, Starosta AL, Virumae K, Atkinson GC, Tenson T, et al (2012) Lys34 of translation elongation factor EF-P is hydroxylated by YfcM. Nat Chem Biol 8: 695–697 doi:10.1038/nchembio.1001 22706199 10.1038/nchembio.1001

[17] Bullwinkle TJ, Zou SB, Rajkovic A, Hersch SJ, Elgamal S, et al (2013) (R)-beta-lysine-modified elongation factor P functions in translation elongation. J Biol Chem 288: 4416–4423. 23277358 10.1074/jbc.M112.438879 PMC3567691

[18] Gutierrez E, Shin BS, Woolstenhulme CJ, Kim JR, Saini P, et al (2013) eIF5A promotes translation of polyproline motifs. Mol Cell 51: 35–45. 23727016 10.1016/j.molcel.2013.04.021 PMC3744875

[19] Park MH (2006) The post-translational synthesis of a polyamine-derived amino acid, hypusine, in the eukaryotic translation initiation factor 5A (eIF5A). J Biochem 139: 161–169. 16452303 10.1093/jb/mvj034 PMC2494880

[20] Ingolia NT, Brar GA, Rouskin S, McGeachy AM, Weissman JS (2012) The ribosome profiling strategy for monitoring translation in vivo by deep sequencing of ribosome-protected mRNA fragments. Nat Protoc 7: 1534–1550. 22836135 10.1038/nprot.2012.086 PMC3535016

[21] Ingolia NT, Ghaemmaghami S, Newman JR, Weissman JS (2009) Genome-wide analysis in vivo of translation with nucleotide resolution using ribosome profiling. Science 324: 218–223. 19213877 10.1126/science.1168978 PMC2746483

[22] Oh E, Becker AH, Sandikci A, Huber D, Chaba R, et al (2011) Selective ribosome profiling reveals the cotranslational chaperone action of trigger factor in vivo. Cell 147: 1295–1308. 22153074 10.1016/j.cell.2011.10.044 PMC3277850

[23] Lorenz R, Bernhart SH, Höner Zu Siederdissen C, Tafer H, Flamm C, et al (2011) ViennaRNA Package 2.0. Algorithms Mol Biol 6: 26. 22115189 10.1186/1748-7188-6-26 PMC3319429

[24] Fredrick K, Ibba M (2010) How the sequence of a gene can tune its translation. Cell 141: 227–229. 20403320 10.1016/j.cell.2010.03.033 PMC2866089

[25] Cruz-Vera LR, Magos-Castro MA, Zamora-Romo E, Guarneros G (2004) Ribosome stalling and peptidyl-tRNA drop-off during translational delay at AGA codons. Nucleic Acids Res 32: 4462–4468. 15317870 10.1093/nar/gkh784 PMC516057

[26] Ramu H, Vazquez-Laslop N, Klepacki D, Dai Q, Piccirilli J, et al (2011) Nascent peptide in the ribosome exit tunnel affects functional properties of the A-site of the peptidyl transferase center. Mol Cell 41: 321–330. 21292164 10.1016/j.molcel.2010.12.031

[27] Corcoran CP, Podkaminski D, Papenfort K, Urban JH, Hinton JC, et al (2012) Superfolder GFP reporters validate diverse new mRNA targets of the classic porin regulator, MicF RNA. Mol Microbiol 84: 428–445. 22458297 10.1111/j.1365-2958.2012.08031.x

[28] Urban JH, Vogel J (2009) A green fluorescent protein (GFP)-based plasmid system to study post-transcriptional control of gene expression in vivo. Methods Mol Biol 540: 301–319. 19381569 10.1007/978-1-59745-558-9_22

[29] Pedelacq JD, Cabantous S, Tran T, Terwilliger TC, Waldo GS (2006) Engineering and characterization of a superfolder green fluorescent protein. Nat Biotechnol 24: 79–88. 16369541 10.1038/nbt1172

[30] Wilson DN, Beckmann R (2011) The ribosomal tunnel as a functional environment for nascent polypeptide folding and translational stalling. Curr Opin Struct Biol 21: 274–282. 21316217 10.1016/j.sbi.2011.01.007

[31] Tanner DR, Cariello DA, Woolstenhulme CJ, Broadbent MA, Buskirk AR (2009) Genetic identification of nascent peptides that induce ribosome stalling. J Biol Chem 284: 34809–34818. 19840930 10.1074/jbc.M109.039040 PMC2787343

[32] Nakatogawa H, Ito K (2002) The ribosomal exit tunnel functions as a discriminating gate. Cell 108: 629–636. 11893334 10.1016/s0092-8674(02)00649-9

[33] Bhushan S, Hoffmann T, Seidelt B, Frauenfeld J, Mielke T, et al (2011) SecM-stalled ribosomes adopt an altered geometry at the peptidyl transferase center. PLoS Biol 9: e1000581. 21267063 10.1371/journal.pbio.1000581 PMC3022528

[34] Dana A, Tuller T (2012) Determinants of translation elongation speed and ribosomal profiling biases in mouse embryonic stem cells. PLoS Comput Biol 8: e1002755. 23133360 10.1371/journal.pcbi.1002755 PMC3486846

[35] Wolin SL, Walter P (1988) Ribosome pausing and stacking during translation of a eukaryotic mRNA. EMBO J 7: 3559–3569. 2850168 10.1002/j.1460-2075.1988.tb03233.x PMC454858

[36] Rychkova A, Mukherjee S, Bora RP, Warshel A (2013) Simulating the pulling of stalled elongated peptide from the ribosome by the translocon. Proc Natl Acad Sci U S A 110: 10195–10200. 23729811 10.1073/pnas.1307869110 PMC3690858

[37] Wilson DN (2011) Peptides in the ribosomal tunnel talk back. Mol Cell 41: 247–248. 21292157 10.1016/j.molcel.2011.01.017

[38] Cano VS, Jeon GA, Johansson HE, Henderson CA, Park JH, et al (2008) Mutational analyses of human eIF5A-1–identification of amino acid residues critical for eIF5A activity and hypusine modification. FEBS J 275: 44–58. 18067580 10.1111/j.1742-4658.2007.06172.x PMC2536608

[39] Dias CA, Cano VS, Rangel SM, Apponi LH, Frigieri MC, et al (2008) Structural modeling and mutational analysis of yeast eukaryotic translation initiation factor 5A reveal new critical residues and reinforce its involvement in protein synthesis. FEBS J 275: 1874–1888. 18341589 10.1111/j.1742-4658.2008.06345.x PMC5278519

[40] Rossi D, Kuroshu R, Zanelli CF, Valentini SR (2014) eIF5A and EF-P: two unique translation factors are now traveling the same road. Wiley Interdiscip Rev RNA 5: 209–222. 24402910 10.1002/wrna.1211

[41] Boël G, Smith PC, Ning W, Englander MT, Chen B, et al (2014) The ABC-F protein EttA gates ribosome entry into the translation elongation cycle. Nat Struct Mol Biol 21: 143–51 doi: 10.1038/nsmb.2740 24389466 10.1038/nsmb.2740 PMC4101993

[42] Chen B, Boel G, Hashem Y, Ning W, Fei J, et al (2014) EttA regulates translation by binding the ribosomal E site and restricting ribosome-tRNA dynamics. Nat Struct Mol Biol 21: 152–159. 24389465 10.1038/nsmb.2741 PMC4143144

[43] Vila-Sanjurjo A, Schuwirth BS, Hau CW, Cate JH (2004) Structural basis for the control of translation initiation during stress. Nat Struct Mol Biol 11: 1054–1059. 15502846 10.1038/nsmb850

[44] Izutsu K, Wada C, Komine Y, Sako T, Ueguchi C, et al (2001) Escherichia coli ribosome-associated protein SRA, whose copy number increases during stationary phase. J Bacteriol 183: 2765–2773. 11292794 10.1128/JB.183.9.2765-2773.2001 PMC99491

[45] Reuven NB, Deutscher MP (1993) Multiple exoribonucleases are required for the 3′ processing of Escherichia coli tRNA precursors in vivo. FASEB J 7: 143–148. 8422961 10.1096/fasebj.7.1.8422961

[46] Elseviers D, Petrullo LA, Gallagher PJ (1984) Novel E. coli mutants deficient in biosynthesis of 5-methylaminomethyl-2-thiouridine. Nucleic Acids Res 12: 3521–3534. 6427754 10.1093/nar/12.8.3521 PMC318766

[47] Wolfe MD, Ahmed F, Lacourciere GM, Lauhon CT, Stadtman TC, et al (2004) Functional diversity of the rhodanese homology domain: the Escherichia coli ybbB gene encodes a selenophosphate-dependent tRNA 2-selenouridine synthase. J Biol Chem 279: 1801–1809. 14594807 10.1074/jbc.M310442200

[48] Ibba M, Soll D (2000) Aminoacyl-tRNA synthesis. Annu Rev Biochem 69: 617–650. 10966471 10.1146/annurev.biochem.69.1.617

[49] Seong IS, Oh JY, Lee JW, Tanaka K, Chung CH (2000) The HslU ATPase acts as a molecular chaperone in prevention of aggregation of SulA, an inhibitor of cell division in Escherichia coli. FEBS Lett 477: 224–229. 10908725 10.1016/s0014-5793(00)01808-1

[50] Malki A, Caldas T, Abdallah J, Kern R, Eckey V, et al (2005) Peptidase activity of the Escherichia coli Hsp31 chaperone. J Biol Chem 280: 14420–14426. 15550391 10.1074/jbc.M408296200

[51] Baba T, Ara T, Hasegawa M, Takai Y, Okumura Y, et al (2006) Construction of Escherichia coli K-12 in-frame, single-gene knockout mutants: the Keio collection. Mol Syst Biol 2: 2006 0008. 10.1038/msb4100050 PMC1681482 16738554

[52] Datsenko KA, Wanner BL (2000) One-step inactivation of chromosomal genes in Escherichia coli K-12 using PCR products. Proc Natl Acad Sci U S A 97: 6640–6645. 10829079 10.1073/pnas.120163297 PMC18686

[53] Guzman LM, Belin D, Carson MJ, Beckwith J (1995) Tight regulation, modulation, and high-level expression by vectors containing the arabinose PBAD promoter. J Bacteriol 177: 4121–4130. 7608087 10.1128/jb.177.14.4121-4130.1995 PMC177145

[54] Gibson DG, Young L, Chuang RY, Venter JC, Hutchison CA 3rd, et al (2009) Enzymatic assembly of DNA molecules up to several hundred kilobases. Nat Methods 6: 343–345. 19363495 10.1038/nmeth.1318

[55] Li H, Durbin R (2009) Fast and accurate short read alignment with Burrows-Wheeler transform. Bioinformatics 25: 1754–1760. 19451168 10.1093/bioinformatics/btp324 PMC2705234

[56] Letzring DP, Dean KM, Grayhack EJ (2010) Control of translation efficiency in yeast by codon-anticodon interactions. RNA 16: 2516–2528. 20971810 10.1261/rna.2411710 PMC2995412

[57] Crooks GE, Hon G, Chandonia JM, Brenner SE (2004) WebLogo: a sequence logo generator. Genome Res 14: 1188–1190. 15173120 10.1101/gr.849004 PMC419797

