# Supplementary figures and images for: EF-P Dependent Pauses Integrate Proximal and Distal Signals during Translation
Source: PLoS Genet. 2014 Aug 21;10(8):e1004553. doi: 10.1371/journal.pgen.1004553 (PMC4140641; doi:10.1371/journal.pgen.1004553)

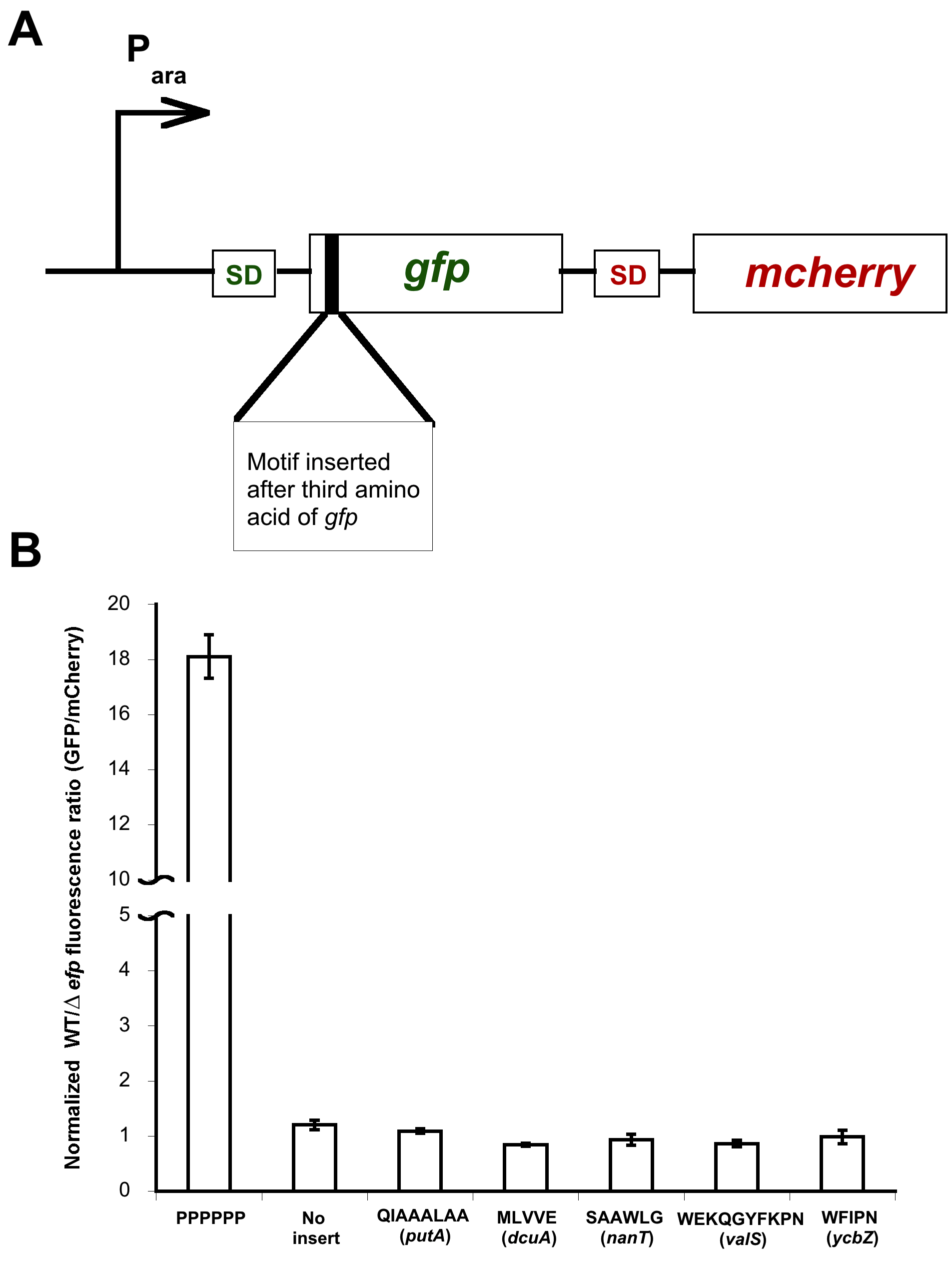

Supplement: Figure S3 — The GFP/mCherry reporter. A) Schematic of the GFP/mCherry reporter: GFP is in a transcriptional fusion to mCherry that has a separate Shine-Dalgarno sequence. mCherry serves as an internal control for variations in transcription and plasmid copy number [11]. Tested motifs were inserted in-frame at the fourth codon of gfp [56]. Fluorescence ratio (GFP/mCherry) is measured for WT and Δefp strains harboring the tested plasmid. The fold difference in fluorescence ratios between WT and Δefp strains is then normalized to the values obtained from a no insert control. B) The reporter construct with the 5 non-PPX motifs described in Table S2. The five motifs have fluorescence ratios lower than no insert, PPPPPP is the positive control. (TIF) [file pgen.1004553.s003.tif]

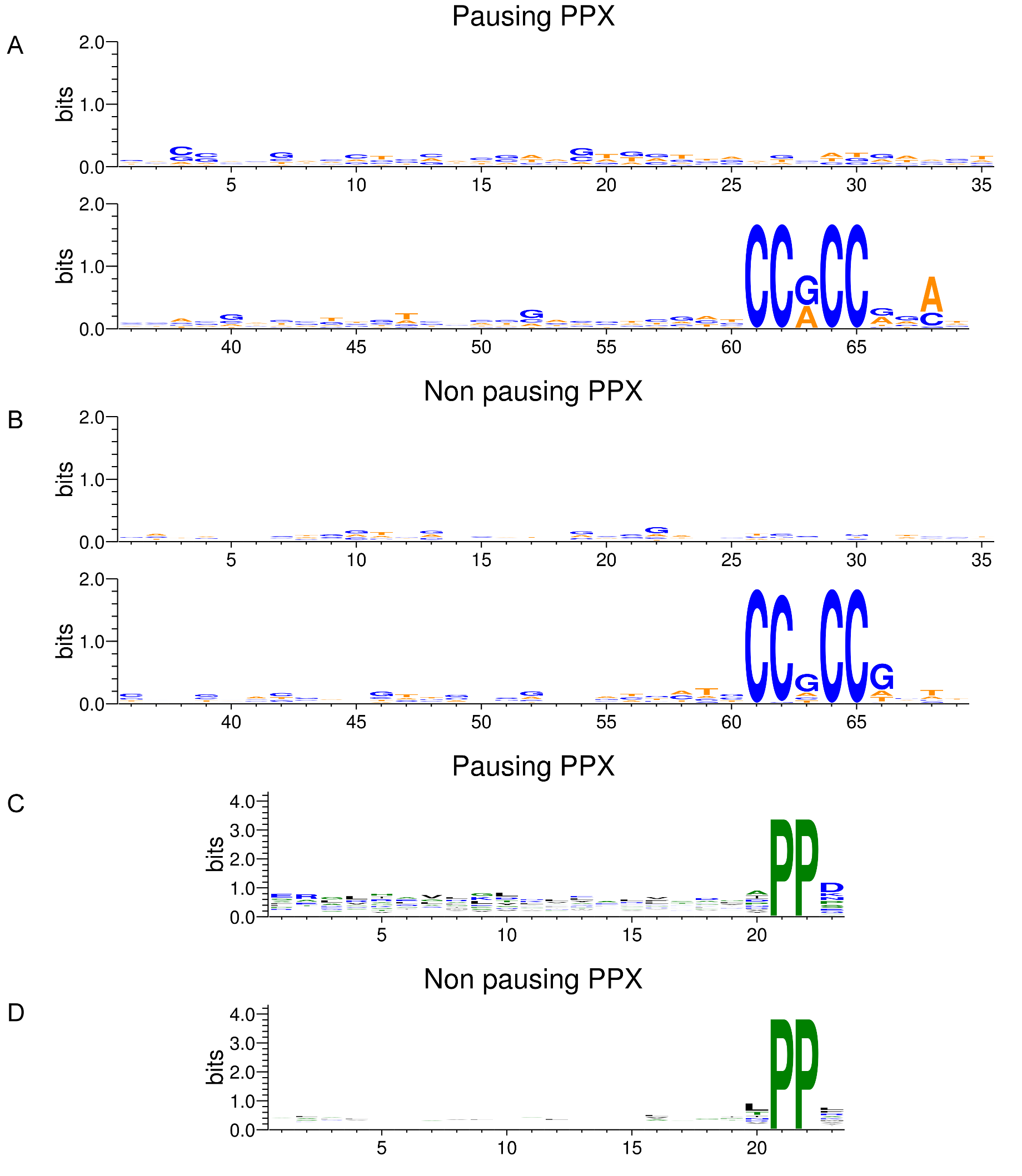

Supplement: Figure S4 — Alignment of sequences from genes with pausing or non-pausing PPX. Figure shows a Logo representation [57] of alignments of nucleotide (A and B) or amino acid (C and D) sequences of pausing (A and C) or non pausing (B and D) PPX containing genes. PPX sequences were manually aligned together with the first 20 upstream codons/amino acids. No gaps were introduced in the sequence. (TIF) [file pgen.1004553.s004.tif]

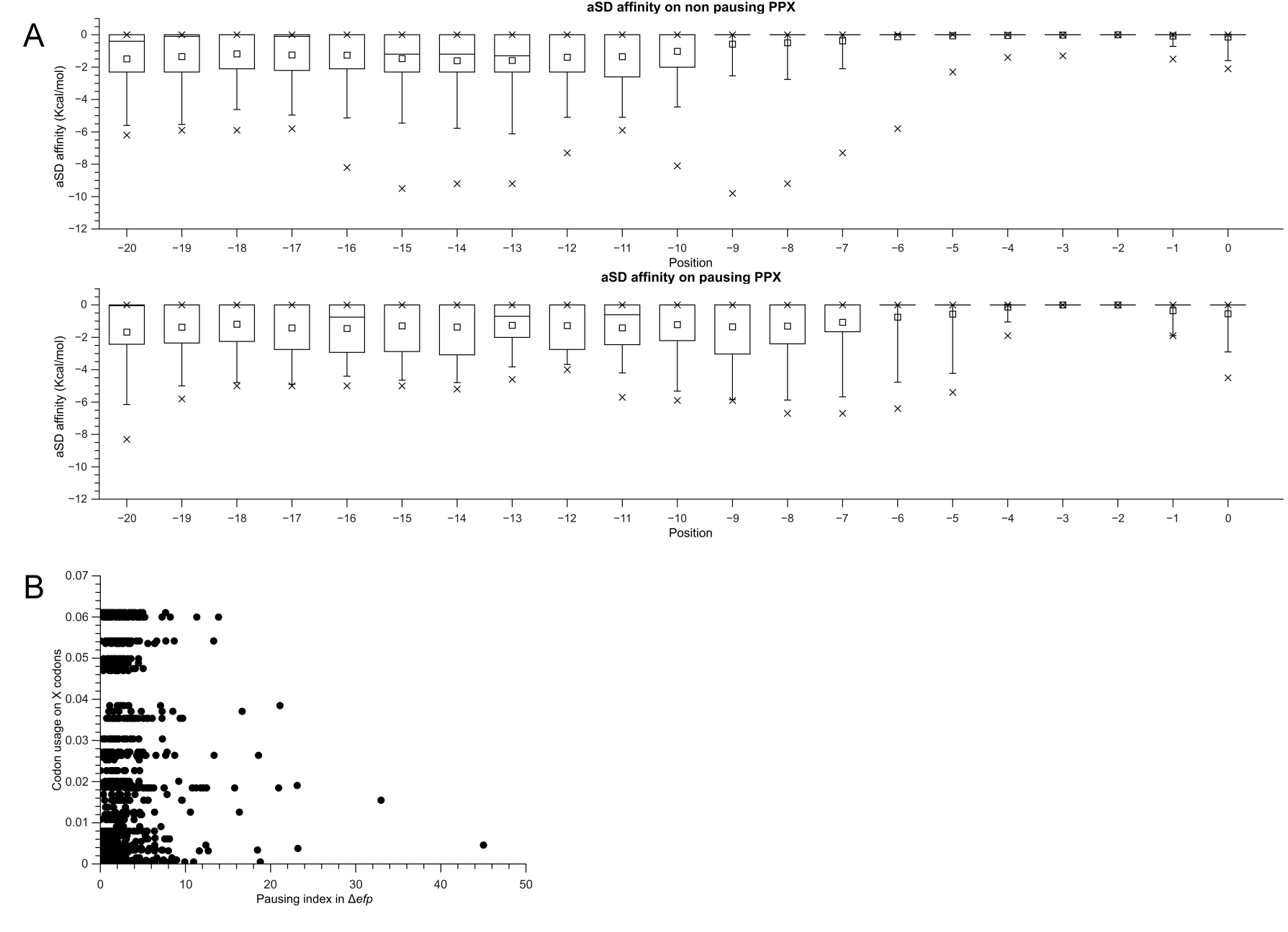

Supplement: Figure S5 — Correlations between mRNA sequence and pause strength. A) Figure shows estimated affinity for ribosomal aSD sequence in sequences upstream of PPX in pausing and non-pausing genes. Position o correspond to the third position of the X codon at PPX (average as a small square, median as the middle line of the box, box limits represent 25th and 75th percentiles, higher and lower values are marked with an “x” symbol). B) Comparison of codon usage for all PPX sequences and their corresponding ribosome occupancies in the strain. Values correspond to average of two biological replicates and are normalized by the average occupancies of the corresponding genes. (TIF) [file pgen.1004553.s005.tif]

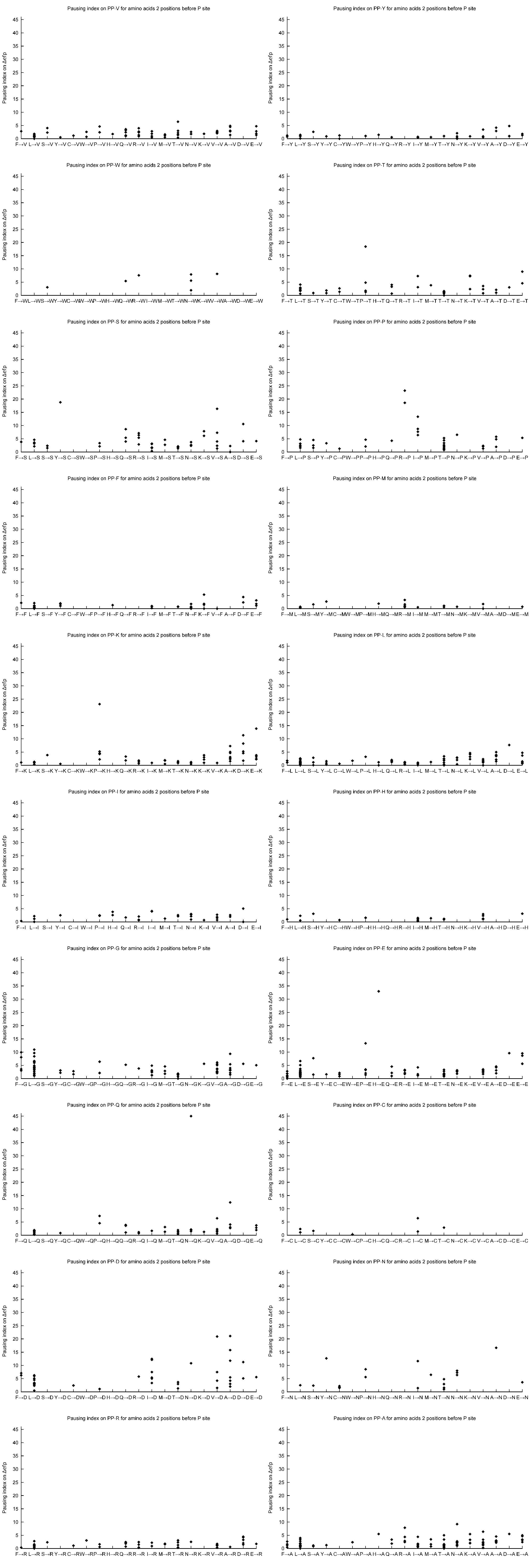

Supplement: Figure S7 — PPX to gene occupancy ratios for all ZPPX combinations found on E. coli. PPX to gene occupancy ratios for all possible amino acid combinations between the last amino of PPX and the one that is immediately upstream of it was plotted. One graph was made for each PPX. (TIF) [file pgen.1004553.s007.tif]

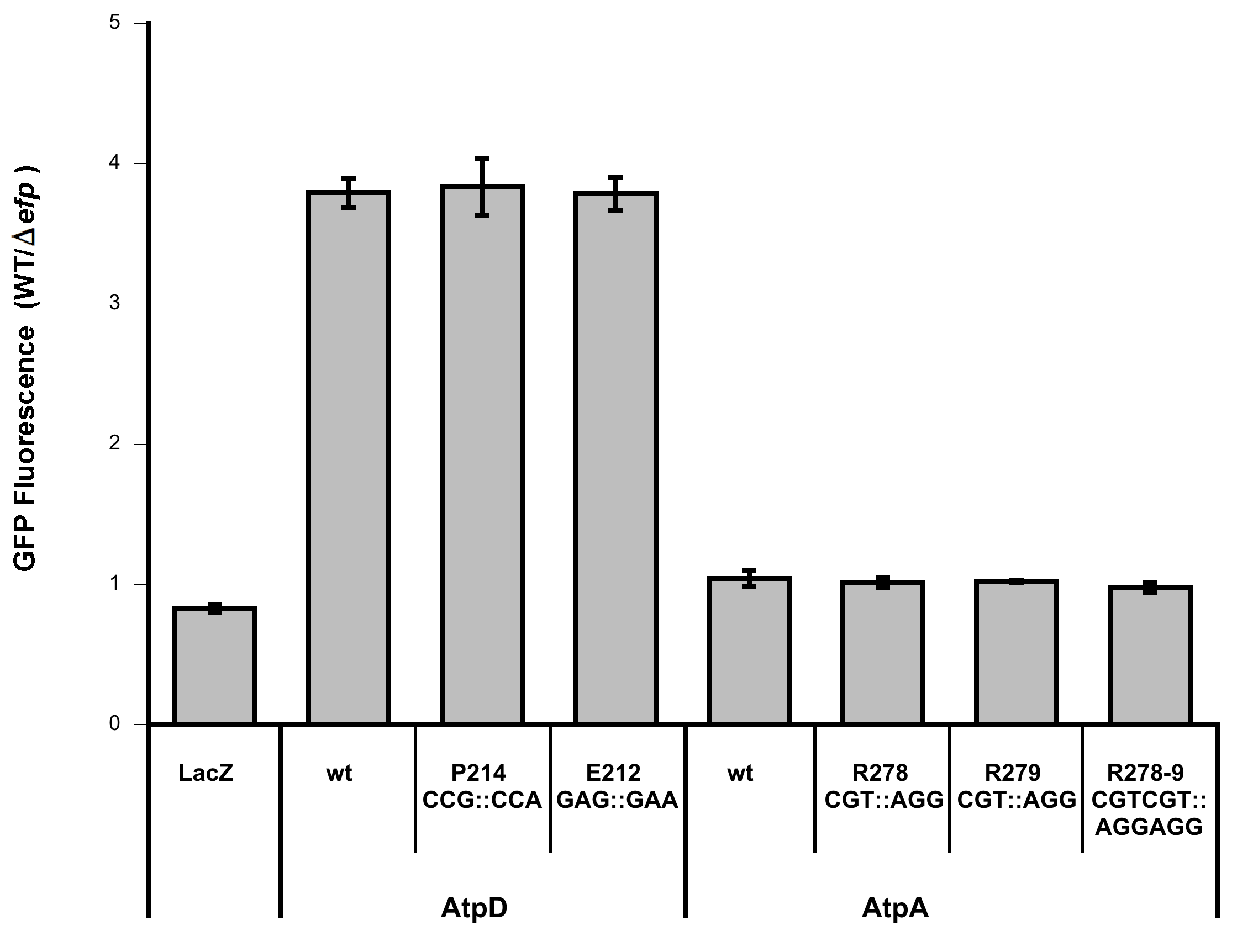

Supplement: Figure S8 — Altering anti-Shine Dalgarno sequence binding or Pro214 codon does not affect expression of AtpD or AtpA. Fluorescence ratios comparing expression of codon mutations in AtpD- and AtpA-GFP fusion constructs in pXG10sf maintaining amino acid sequence while altering binding to the anti-Shine Dalgarno sequence of 16s rRNA, or altering the second proline codon of the AtpD PPG motif (CCG) to the CCA pro codon in that position in AtpA. LacZ and unmodified (wt) AtpD and AtpA constructs from Figure 4 are included for comparison. Ratios show WT/Δefp (Salmonella Typhimurium) for GFP fluorescence at 10 hours post-inoculation normalized to optical density (600 nm). The mean of at least three biological replicates is shown and error bars indicate one standard deviation. (TIF) [file pgen.1004553.s008.tif]

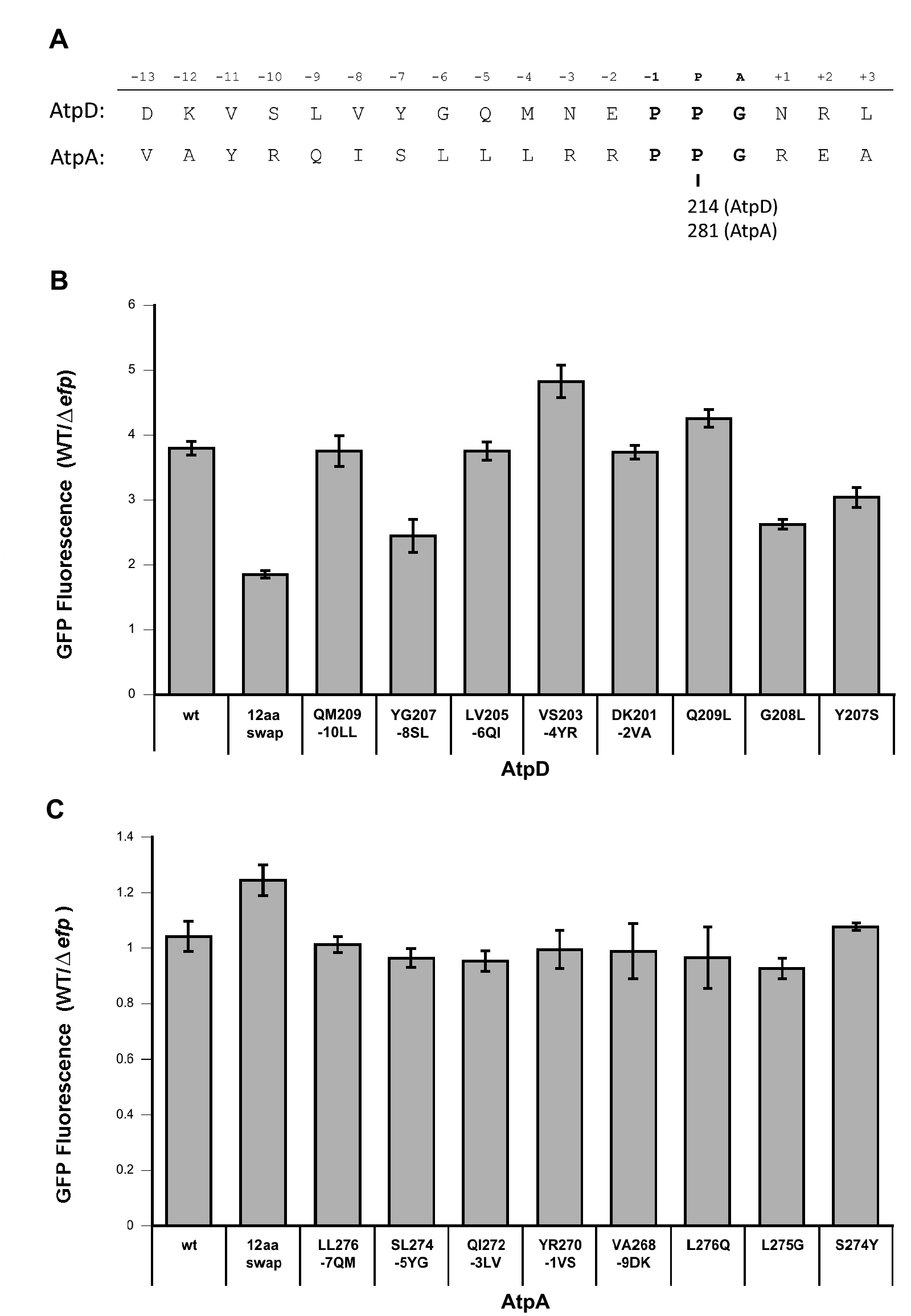

Supplement: Figure S9 — Extended mutagenesis of residues upstream of AtpD and AtpA PPG motif. A. As in Figure 5 , showing sequence (Salmonella Typhimurium) of AtpD and AtpA in proximity to their PPG motifs (bold). The relative position when the PPG glycine occupies the A site is shown above. The amino acid position of the second proline of the PPG motif in each protein is indicated below. B. Constructs generated in addition to those shown in Figure 5 . Values are fluorescence ratios comparing expression of plasmid-borne AtpD-GFP translational fusions in wild-type (WT) and efp mutant Salmonella. Unmodified (wt) and 12aa swap construct from Figure 5 are shown for comparison. Ratios show WT/Δefp for GFP fluorescence at 10 hours post-inoculation normalized to optical density (600 nm). The mean of at least three biological replicates is shown and error bars indicate one standard deviation. C. As in B, but with AtpA-GFP translational fusion constructs with swap-in of AtpD upstream sequence. (TIF) [file pgen.1004553.s009.tif]

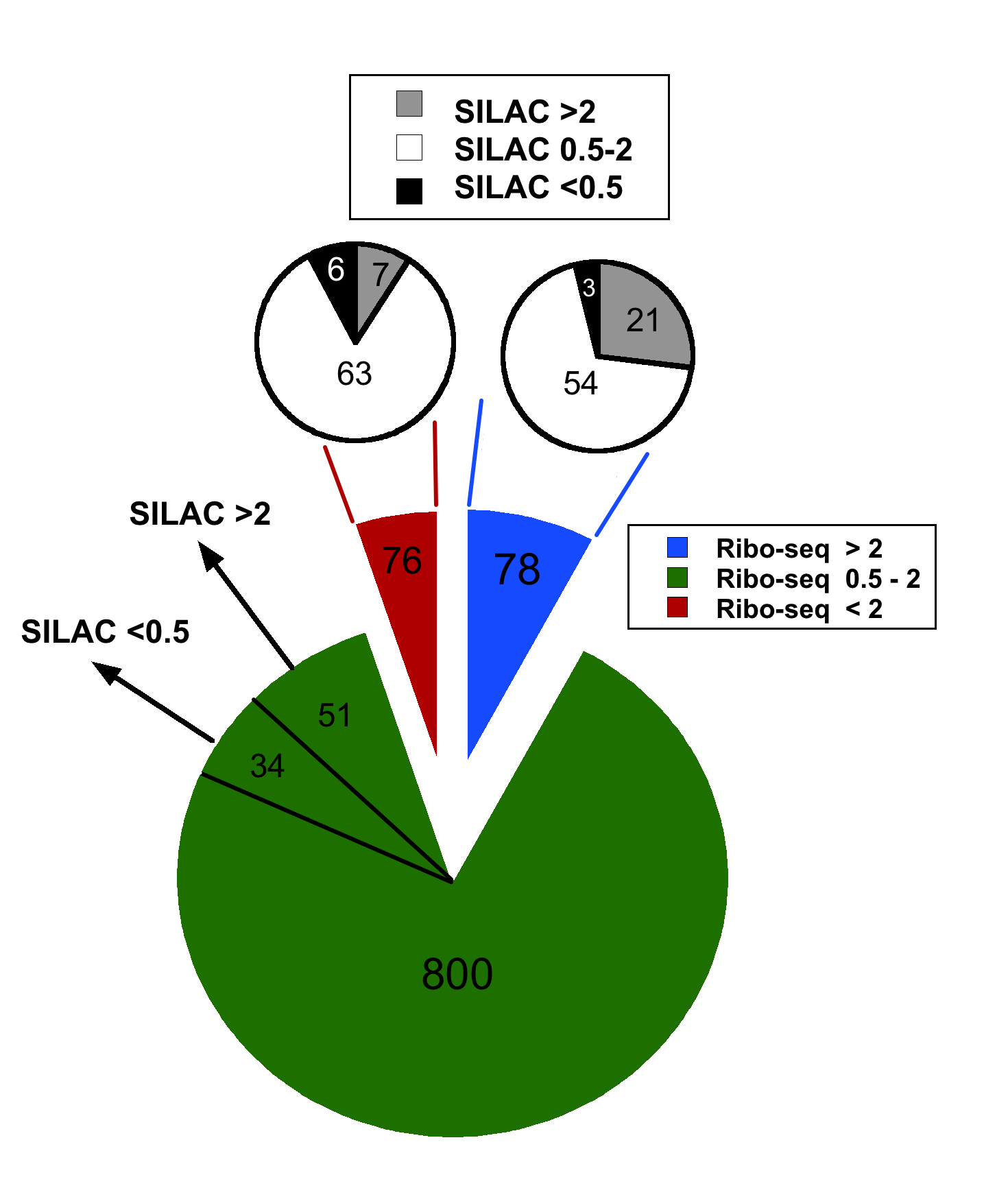

Supplement: Figure S10 — Comparison of SILAC and rib-seq data. Pie chart comparing proteins identified in our ribo-seq experiment (cutoff 70 footprint reads/gene) and also present in Peil et al., SILAC dataset [10]. Ribo-seq data (ratio between Δefp and WT footprints/gene) was compared with SILAC data (protein abundance ratio between Δefp and WT). In 77% (800 out of 1039) of the proteins, the ratio for both datasets was between 0.5–2. For 7.5% of the genes, there was more than 2 fold higher total footprints/gene in Δefp vs. WT, about one fourth of them also had above 2 folds more protein abundance in Δefp vs. WT. While 7.3% of the genes had less had less than 0.5 fold footprints/gene in Δefp vs. WT, about one tenth of them also had less than 0.5 fold protein abundance in Δefp vs. WT. (TIF) [file pgen.1004553.s010.tif]
