# Supplementary material for: EF-P Dependent Pauses Integrate Proximal and Distal Signals during Translation
Source: PLoS Genet. 2014 Aug 21;10(8):e1004553. doi: 10.1371/journal.pgen.1004553 (PMC4140641; doi:10.1371/journal.pgen.1004553)
Supplement: Table S1 — List of PPX-containing proteins that were identified by SILAC [10] to be 3-fold or more abundant in the wild-type vs. Δefp strain and their corresponding pausing index from ribo-seq. (DOC) [file pgen.1004553.s011.doc]

**Table S1**. List of PPX-containing proteins that were identified by SILAC [Peil, 2013 #22] to be 3-fold or more abundant in the wild-type vs. ∆*efp* strain and their corresponding pausing index from ribo-seq

| Gene | PPX at pause | SILAC average *E. coli* WT/∆*efp**1 | Upstream sequence | Pausing index *^1^ | | |
| --- | --- | --- | --- | --- | --- | --- |
|  |  |  |  | WT | ∆*efp* | Complemented  (∆*efp* pEF-P) |
| atpD | PPG | 5.18 | EMTDSNVIDKVSLVYGQMNE | 0.62 | 5.04 | 3.17 |
| cadA | PPL | 6.78 | AEDIANKIKQTTDEYINTIL | NA | NA | NA |
|  | PPG |  | EEVYLDEMVGRINANMILPY |  |  |  |
| hrpA | PPA | 3.73 | AQMLKVENVQQAWQQWINKL | 0.00 | 0.00 | 0.00 |
|  | PPK |  | YTGARNARFSIFPGSGLFKK | 1.17 | 3.80 | 2.39 |
| *lepA* | PPE | 3.59 | SAKTGVGVQDVLERLVRDIP | 2.12 | 13.3 | 5.1 |
|  | PPP |  | CSAKTGVGVQDVLERLVRDI | 2.17 | 13.35 | 5.18 |
| malQ | PPD | 3.49 | GGAETWCDRELYCLKASVGA | 2.12 | 4.20 | 4.97 |
|  | PPM |  | KASVGAPPDILGPLGQNWGL | 0.23 | 0.69 | 0.38 |
| mcrB | PPM | 3.01 | MLDNIINDYKLIFNSGKSVI | 1.20 | 0.52 | 1.34 |
|  | PPG |  | TTIETILKRLTIKKNIILQG | 0.00 | 1.63 | 0.20 |
| nadC | PPR | 3.42 | -------------------M | 1.99 | 1.79 | 1.81 |
| pdxB | PPR | 3.36 | VGRRLQARLEALGIKTLLCD | 0.94 | 4.23 | 3.60 |
| pncA | PPR | 4.04 | -------------------M | 0.35 | 1.59 | 2.43 |
| pyrC | PPV | 5.32 | VVPYTSEIYGRAIVMPNLAA | 0.35 | 2.77 | 1.45 |
| rnb | PPQ | 7.01 | ATEKGFGFLEVDAQKSYFIP | 1.24 | 7.47 | 2.93 |
| ycgR | PPT | 6.58 | TVEQLQQSEYLQLPAFITVP | 9.19 | 1.29 | 1.10 |
|  | PPY |  | PPTLWFVQRRRYFRISAPLH | 0.00 | 1.47 | 1.42 |
| ygdH | PPE | 3.28 | ENFDINVLRRERGVKLELIN | 0.15 | 3.13 | 1.09 |
|  | PPN |  | QRYKDSRFIGMTEPSIIAAE | 0.46 | 3.60 | 3.02 |
| yhgF | PPK | 3.48 | RGRNEGVLQLSLNADPQFDE | 0.61 | 3.81 | 3.00 |
| yjjK | PPG | 3.47 | FEELNSTEYQKRNETNELFI | 0.23 | 3.10 | 1.47 |
|  | PPK |  | -----MAQFVYTMHRVGKVV | 0.40 | 0.88 | 0.62 |
| ytfM | PPP | 3.15 | IREGLKALGYYQPTIEFDLR | 1.23 | 23.2 | 7.92 |
|  | PPK |  | REGLKALGYYQPTIEFDLRP | 1.4 | 23.11 | 8.04 |

*^1^:These values from SILAC by Peil *et al.,* [Peil, 2013 #22]
