# Supplementary material for: EF-P Dependent Pauses Integrate Proximal and Distal Signals during Translation
Source: PLoS Genet. 2014 Aug 21;10(8):e1004553. doi: 10.1371/journal.pgen.1004553 (PMC4140641; doi:10.1371/journal.pgen.1004553)
Supplement: Table S2 — List of EF-P dependent pauses that do not contain a PPX sequence. (DOC) [file pgen.1004553.s012.doc]

**Table S2**: List of EF-P dependent pauses that do not contain a PPX sequence

| Gene *^1^ | Sequences examined at pause *^2^ | Upstream sequences | Pausing index *^3^ | | | SILAC average E. coli WT/ ∆*efp* *4 |
| --- | --- | --- | --- | --- | --- | --- |
|  |  |  | WT | ∆*efp* | Complemented  (∆*efp* pEF-P) |  |
| *dcuA* *^5^ | MLVVE | -------------------- | 12.28 | 20.2 | 10.18 | NA |
|  |  |  |  |  |  |  |
| *nanT* | SAAWLG  AQWRAFSAAW | TTTQNIPWYRHLNRAQWRAF  MSTTTQNIPWYRHLNR | 6.89 | 13.6 | 2.01 | NA |
|  |  |  |  |  |  |  |
| *putA* | QIAAALAA  IFTGQIAAAL | PLGPVVCISPWNFPLAIFTG  ETHRPLGPVVCISPWNFPLA | 0.58 | 18.92 | 8.79 | 0.26 |
|  |  |  |  |  |  |  |
| *valS* | WEKQGYFKPN  EHWEKQGYFK | MEKTYNPQDIEQPLY  MEKTYNPQDIEQPLYEH | 0.68 | 11.12 | 2.77 | 5.75 |
|  |  |  |  |  |  |  |
| *ycbZ* | WFIPN#  LRWLNWFIPN# | AQASQQEGRHRFPWPLRWLN  IQERIAQASQQEGRHRFPWP | 4.96 | 11.91 | 8.27 | 1.14 |

*^1^: Only genes where both samples from the ∆*efp* strain show a pause and where the complemented strain present a decrease of ribosome occupancies at the pausing site were included.

*^2^: “#” represents a stop codon.

*^3^: Values correspond to averages of two independent samples.

*^4^: These values from SILAC by Peil *et al.,*

*^5^: *dcuA* pausing site includes the translation start codon and thus, it doesn't have any upstream amino acid sequence.
