# Supplementary material for: EF-P Dependent Pauses Integrate Proximal and Distal Signals during Translation
Source: PLoS Genet. 2014 Aug 21;10(8):e1004553. doi: 10.1371/journal.pgen.1004553 (PMC4140641; doi:10.1371/journal.pgen.1004553)
Supplement: Table S3 — List of PPX sequences that do not produce a translation pause. (DOC) [file pgen.1004553.s013.doc]

**Table S3**. List of PPX sequences that do not produce a translation pause *^1^.

| Gene *^3^ | PPX at pause | Upstream sequence | Pausing index *^2^ | | |
| --- | --- | --- | --- | --- | --- |
|  |  |  | WT | ∆*efp* | Complemented  (∆*efp* pEF-P) |
| *yaaA* | PPQ | TLPELLDNSQQLIHEARKLT | 0.27 | 0.45 | 0.67 |
| *frr* | PPL | MASDLGLNPNSAGSDIRVPL | 0.65 | 0.42 | 0.58 |
| *lpxA* | PPY | FCIIGAHVMVGGCSGVAQDV | 0.15 | 0.56 | 0.51 |
| *gpt* | PPI | VDIPQDTWIEQPWDMGVVFV | 0.75 | 0.70 | 0.68 |
| *tesB* | PPV | PAPDGLPSETQIAQSLAHLL | 0.50 | 0.88 | 0.69 |
| *aroG* | PPV | ---MNYQNDDLRIKEIKELL | 0.16 | 0.67 | 0.41 |
| *ybiB* | PPL | QAQAKLDEHQPVFMPVGAFC | 0.68 | 0.58 | 0.95 |
| *pflA* | PPK | ELGKHKWVAMGEEYKLDGVK | 0.58 | 0.93 | 0.84 |
| *rlmI* | PPA | VAVRKKEGMELTQGPVTGEL | 0.24 | 0.93 | 0.21 |
| *cbpA* | PPK | KGLVSKKQTGDLYAVLKIVM | 0.62 | 0.49 | 1.22 |
| *ycdY* | PPY | DMTQVSADYNALFIGDECAV | 0.31 | 0.89 | 0.55 |
| *flgA* | PPR | RGGKLEAGNVKLKRGRLDTL | 0.39 | 0.89 | 0.29 |
| *ndh* | PPR | PDIYAIGDCASCPRPEGGFV | 0.21 | 0.32 | 0.56 |
| *phoP* | PPF | ARMQALMRRNSGLASQVISL | 0.46 | 0.51 | 0.70 |
| *galU* | PPH | EAMLEKRVKRQLLDEVQSIC | 1.07 | 0.71 | 0.74 |
| *topA* | PPK | KMGIRTASTGVFLGCSGYAL | 0.52 | 0.35 | 0.92 |
| *tyrR* | PPL | QKGMFREDLYYRLNVLTLNL | 0.17 | 0.89 | 0.90 |
| *rstA* | PPA | DMNHILALEMGACDYILKTT | 0.08 | 0.34 | 0.09 |
| *yniA* | PPR | PKVWAVGADRDYSFLVMDYL | 1.19 | 0.36 | 0.16 |
| *nadE* | PPI | PQQVARTIENWYLKTEHKRR | 0.18 | 0.83 | 0.37 |
| *yeaK* | PPF | ASLASPAEVDELTGCVFGAI | 0.06 | 0.20 | 0.07 |
| *aspS* | PPH | NEEEQREKFGFLLDALKYGT | 0.32 | 0.89 | 0.84 |
| *gyrA* | PPH | IPNLLVNGSSGIAVGMATNI | 0.31 | 0.61 | 0.70 |
| *glpT* | PPL | KERGGIVSVWNCAHNVGGGI | 0.11 | 0.04 | 0.00 |
| *glpT* | PPC | SIAVMFVLLFLCGWFQGMGW | 0.05 | 0.27 | 0.20 |
| *glpT* | PPI | ILVALFAFAMMRDTPQSCGL | 0.12 | 0.02 | 0.02 |
| *yfbV* | PPA | ALSLPMQGLWWLGKRSVTPL | 0.73 | 0.59 | 0.80 |
| *pta* | PPL | QDEVDGLVSGAVHTTANTIR | 1.03 | 0.30 | 0.80 |
| *prmB* | PPY | IRSDLFRDLPKVQYDLIVTN | 0.42 | 0.87 | 0.56 |
| *sixA* | PPM | VISHLPLVGYLVAELCPGET | 1.33 | 0.57 | 0.77 |
| *maeB* | PPL | KKMARAPMILALANPEPEIL | 0.32 | 0.81 | 0.67 |
| *dapE* | PPF | LAFAGHTDVVPPGDADRWIN | 0.66 | 0.69 | 0.64 |
| *srmB* | PPA | LEALQDKGFTRPTAIQAAAI | 0.01 | 0.57 | 0.32 |
| *ung* | PPQ | YFLNTLQTVASERQSGVTIY | 0.38 | 0.83 | 1.04 |
| *sdaC* | PPP | YSVAITNTVESFMSHQLGMT | 1.65 | 0.75 | 1.11 |
| *sdaC* | PPR | SVAITNTVESFMSHQLGMTP | 1.46 | 0.59 | 0.86 |
| *ygfZ* | PPR | --------------MAFTPF | 0.57 | 0.35 | 0.18 |
| *gcvP* | PPV | ASRNKRFTSYIGMGYTAVQL | 0.44 | 0.80 | 0.84 |
| *speA* | PPM | YIDGDGIATTMPMPEYDPEN | 0.47 | 0.72 | 0.32 |
| *hybO* | PPV | MGLSSKAAAEMAESVTNPQR | 0.21 | 0.92 | 0.87 |
| *yqhD* | PPR | HSAHVQPVFAVLDPVYTYTL | 0.04 | 0.75 | 0.35 |
| *parE* | PPL | ALFVKHFRALVKHGHVYVAL | 0.14 | 0.59 | 0.13 |
| *yqiC* | PPT | ISELENRSTEIKKQPDPETL | 0.09 | 0.59 | 0.40 |
| *gltB* | PPF | KLLETAEPHPGKALYCTENN | 0.19 | 0.30 | 0.20 |
| *gltB* | PPI | SSQPRIIYDYFRQQFAQVTN | 0.17 | 0.92 | 0.38 |
| *aroK* | PPR | EKQLARTQRDKKRPLLHVET | 0.09 | 0.42 | 0.42 |
| *pck* | PPV | SKAGHATKVIFLTADAFGVL | 0.69 | 0.87 | 0.43 |
| *yhiR* | PPY | FQQLKAKLPPVSRRGLILID | 0.18 | 0.99 | 0.22 |
| *yibT* | PPR | DKAVDFMASSQAFREYLKKL | 0.34 | 0.33 | 0.23 |
| *lldD* | PPF | ---MIISAASDYRAAAQRIL | 0.42 | 0.63 | 0.44 |
| *dfp* | PPF | AAARRGANVTLVSGPVSLPT | 1.00 | 0.70 | 0.71 |
| *ibpA* | PPY | GFDRLFNHLENNQSQSNGGY | 1.03 | 0.87 | 1.40 |
| *ilvE* | PPF | YISEGAGENLFEVKDGVLFT | 0.70 | 0.77 | 0.20 |
| *rho* | PPK | PSQIRRFNLRTGDTISGKIR | 0.64 | 0.64 | 0.67 |
| *fdhE* | PPL | IRIIPQDELGSSEKRTADMI | 0.04 | 0.46 | 0.15 |
| *cytR* | PPM | MLLLGSRLPFDASIEEQRNL | 0.33 | 0.72 | 0.51 |
| *cytR* | PPT | GDFTFEAGSKAMQQLLDLPQ | 0.44 | 0.68 | 0.87 |
| *rplK* | PPA | PIPVVITVYADRSFTFVTKT | 0.76 | 0.41 | 0.43 |
| *malE* | PPK | AYPIAVEALSLIYNKDLLPN | 0.14 | 0.47 | 0.27 |
| *malK* | PPA | GLETITSGDLFIGEKRMNDT | 0.83 | 0.73 | 0.53 |
| *ssb* | PPM | FSGGAQSRPQQSAPAAPSNE | 0.52 | 0.71 | 0.82 |
| *ytfB* | PPM | KPTLEKVWHAPDNFRFMDPL | 0.85 | 0.47 | 0.58 |
| *fklB* | PPF | WELTIPQELAYGERGAGASI | 0.88 | 0.84 | 0.97 |
| *nrdD* | PPL | HLDVEKKVNPYDKIDFEAPY | 0.39 | 0.24 | 0.37 |
| *yjjK* | PPK | -----MAQFVYTMHRVGKVV | 0.40 | 0.88 | 0.62 |
| *rob* | PPL | FAQTPALYRRSPEWSAFGIR | 0.28 | 0.78 | 0.56 |

*^1^: Only PPX sequences that present ribosome densities equal or lower than the gen average were considered in this list. Also genes where the PPX sequence was immediately down stream of a translation pause were eliminated from the list to prevent the inclusion of genes where a pausing PPX was masked by the inability of ribosomes to pass a previous pause.

*^2^: Values correspond to averages of two independent samples

*^3^: Genes with a PPPX pattern are introduced twice to account for the ribosome density at P_1_P_2_P_3_ and P_2_P_3_X
