# Supplementary material for: EF-P Dependent Pauses Integrate Proximal and Distal Signals during Translation
Source: PLoS Genet. 2014 Aug 21;10(8):e1004553. doi: 10.1371/journal.pgen.1004553 (PMC4140641; doi:10.1371/journal.pgen.1004553)
Supplement: Table S4 — Combinations of amino acids at A site (X, #10) and 2 positions upstream the P site (Z) in pausing and non-pausing ZPPX sequences. (DOC) [file pgen.1004553.s014.doc]

**Table S4.** Combinations of amino acids at A site (X, #10) and 2 positions upstream the P site (Z) in pausing and non-pausing ZPPX sequences.

| **{-2} (Z) *^1^** | **{A site} (X, #10) *^1^** | **No-pause cases** | **Identity -2** | **Identity A site** | **Pause cases** | **Identity -2** | **Identity A site** |
| --- | --- | --- | --- | --- | --- | --- | --- |
| Hydrophobic | Acidic | 0 |  |  | 6 | AAIAVI | DDDDDD |
| Basic | Acidic | 0 |  |  | 1 | H | E |
| Acidic | Acidic | 0 |  |  | 1 | D | D |
| Amide | Acidic | 0 |  |  | 1 | N | D |
| Hydrophobic | Amide | 0 |  |  | 4 | AAII | NQN |
| OH | Amide | 2 | TY | QQ | 1 | Y | N |
| Amide | Amide | 0 |  |  | 1 | N | Q |
| Hydrophobic | Aromatic | 4 | LILI | FFFF | 0 |  |  |
| OH | Aromatic | 2 | TT | FF | 0 |  |  |
| Amide | Aromatic | 3 | NNN | YFF | 0 |  |  |
| Hydrophobic | Basic | 9 | MLVLLILLV | KRRKRHRRK | 0 |  |  |
| Basic | Basic | 2 | KR | KK | 0 |  |  |
| Acidic | Basic | 0 |  |  | 2 | DE | KK |
| OH | Basic | 2 | TT | HR | 0 |  |  |
| Amide | Basic | 1 | N | K | 0 |  |  |
| Cys | Basic | 1 | C | H | 0 |  |  |
| Aromatic | Basic | 1 | F | R | 0 |  |  |
| Hydrophobic | Glycine | 0 |  |  | 1 | L | G |
| Hydrophobic | Hydrophobic | 17 | LVLLLLILLLILLLILL | LIVVALLIALAVLVLMM | 0 |  |  |
| Acidic | Hydrophobic | 1 | E | M | 0 |  |  |
| Basic | Hydrophobic | 4 | RRRR | ILVL | 0 |  |  |
| Cys | Hydrophobic | 1 | C | L | 0 |  |  |
| OH | Hydrophobic | 5 | TTTTY | AMAAL | 0 |  |  |
| Amide | Hydrophobic | 2 | NN | MI | 0 |  |  |
| Acidic | OH | 1 | D | Y | 1 | D | S |
| OH | OH | 1 | Y | Y | 1 | Y | S |
| Amide | OH | 1 | Q | T | 0 |  |  |
| Aromatic | OH | 0 |  |  | 0 |  |  |
| Hydrophobic | OH | 3 | LVV | TYY | 1 | V | S |
| Aromatic | SH | 1 | W | C | 0 |  |  |
| Basic | Pro | 0 |  |  | 2 | RR | P |
| OH | Pro | 1 | T | P | 0 |  |  |
| Hydrophobic | Pro | 0 |  |  | 1 | I | P |

*^1^ Pauses containing PPP pauses are not included in the analysis to prevent errors in interpreting A site and -2 positions.
