# Supplementary material for: EF-P Dependent Pauses Integrate Proximal and Distal Signals during Translation
Source: PLoS Genet. 2014 Aug 21;10(8):e1004553. doi: 10.1371/journal.pgen.1004553 (PMC4140641; doi:10.1371/journal.pgen.1004553)
Supplement: Table S5 — Indexed library PCR primers. (DOCX) [file pgen.1004553.s015.docx]

**Table S5:** Indexed library PCR primers.

| **Forward index (5'→3')** | **Indexed reverse library PCR primer (5'→3')** * |
| --- | --- |
| ACGACT | CAAGCAGAAGACGGCATACGAGAT**AGTCGT**GTGACTGGAGTTCAGACGTGTGCTCTTCCG |
| ATCAGT | CAAGCAGAAGACGGCATACGAGAT**ACTGAT**GTGACTGGAGTTCAGACGTGTGCTCTTCCG |
| CAGCAT | CAAGCAGAAGACGGCATACGAGAT**ATGCTG**GTGACTGGAGTTCAGACGTGTGCTCTTCCG |
| CGACGT | CAAGCAGAAGACGGCATACGAGAT**ACGTCG**GTGACTGGAGTTCAGACGTGTGCTCTTCCG |
| GCAGCT | CAAGCAGAAGACGGCATACGAGAT**AGCTGC**GTGACTGGAGTTCAGACGTGTGCTCTTCCG |
| TACGAT | CAAGCAGAAGACGGCATACGAGAT**ATCGTA**GTGACTGGAGTTCAGACGTGTGCTCTTCCG |
| CTGACG | CAAGCAGAAGACGGCATACGAGAT**CGTCAG**GTGACTGGAGTTCAGACGTGTGCTCTTCCG |
| GCTACG | CAAGCAGAAGACGGCATACGAGAT**CGTAGC**GTGACTGGAGTTCAGACGTGTGCTCTTCCG |
| TTAGGC | CAAGCAGAAGACGGCATACGAGAT**GCCTAA**GTGACTGGAGTTCAGACGTGTGCTCTTCCG |
| TGACCA | CAAGCAGAAGACGGCATACGAGAT**TGGTCA**GTGACTGGAGTTCAGACGTGTGCTCTTCCG |
| ACAGTG | CAAGCAGAAGACGGCATACGAGAT**CACTGT**GTGACTGGAGTTCAGACGTGTGCTCTTCCG |
| GCCAAT | CAAGCAGAAGACGGCATACGAGAT**ATTGGC**GTGACTGGAGTTCAGACGTGTGCTCTTCCG |

* Underlined sequence indicates the reverse complement of the index sequence recognized during Illumina sequencing [Ingolia, 2012 #13].
