# Supplementary material for: EF-P Dependent Pauses Integrate Proximal and Distal Signals during Translation
Source: PLoS Genet. 2014 Aug 21;10(8):e1004553. doi: 10.1371/journal.pgen.1004553 (PMC4140641; doi:10.1371/journal.pgen.1004553)
Supplement: Table S6 — Strains, plasmids and primers used in this study. (DOC) [file pgen.1004553.s016.doc]

**Table S6:** Strains, plasmids and primers used in this study.

**Strains**

| Strain | Genotype or relevant characteristics |
| --- | --- |
| BW25113 | “wild type” E. coli, parental strain used for the Keio collection [Baba, 2006 #1] |
| MW1014 | ∆*efp* of Keio collection with kanamycin cassette removed using FLP recombinase [Hersch, 2013 #11] |
| WN150 | “wild type” *Salmonella* *enterica* serovar Typhimurium strain 14028s [Hersch, 2013 #11] |
| WN1405 | ∆*efp* mutation (deletion of base-pairs 145-424) in WN150 [Hersch, 2013 #11] |

**Plasmids**

| Plasmids | Description |
| --- | --- |
| pBAD30mw700 | pBAD30 plasmid with *gfp* and *mCherry* tandem reporter with their respective Shine-Dalgarno sequences [Hersch, 2013 #11] |
| pBAD30XS | pBAD30mw700 with an xhoI & an speI cloning site at forth codon position of *gfp* |
| pBAD30XS2 | pBAD30XS with PPPPPP inserted between the xhoI and speI sites |
| pBAD30XS3 | pBAD30XS with PPG inserted between the xhoI and speI sites |
| pBAD30XS4 | pBAD30XS with PPN inserted between the xhoI and speI sites |
| pBAD30XS5 | pBAD30XS with GQIAAALAA (putA) inserted between the xhoI and speI sites |
| pBAD30XS25 | pBAD30XS with NPPN inserted between the xhoI and speI sites |
| pBAD30XS27 | pBAD30XS with PPKinserted between the xhoI and speI sites |
| pBAD30XS28 | pBAD30XS with LPPPAK inserted between the xhoI and speI sites |
| pBAD30XS29 | pBAD30XS with PEPPK1c - 1.2 inserted between the xhoI and speI sites |
| pBAD30XS30 | pBAD30XS with PEPPR1 - 2.4 inserted between the xhoI and speI sites |
| pBAD30XS31 | pBAD30XS with PEPPH1 - 1.2 inserted between the xhoI and speI sites |
| pBAD30XS32 | pBAD30XS with PKPPK1a inserted between the xhoI and speI sites |
| pBAD30XS33 | pBAD30XS with PVPPK1a inserted between the xhoI and speI sites |
| pBAD30XS34 | pBAD30XS with PTPPK1a inserted between the xhoI and speI sites |
| pBAD30XS35 | pBAD30XS with PFPPK1a inserted between the xhoI and speI sites |
| pBAD30XS36 | pBAD30XS with LPPL (frr) inserted between the xhoI and speI sites |
| pBAD30XS37 | pBAD30XS with LPPR (flgA) inserted between the xhoI and speI sites |
| pBAD30XS38 | pBAD30XS with KPPK (pflA) inserted between the xhoI and speI sites |
| pBAD30XS39 | pBAD30XS with VPPD (ubiB) inserted between the xhoI and speI sites |
| pBAD30XS40 | pBAD30XS with IPPN ( trmL) inserted between the xhoI and speI sites |
| pBAD30XS41 | pBAD30XS with HPPE (rsxc) inserted between the xhoI and speI sites |
| pBAD30XS42 | pBAD30XS with APPN (fliP) inserted between the xhoI and speI sites |
| pBAD30XS43 | pBAD30XS with RPPPKK inserted between the xhoI and speI sites |
| pBAD30XS44 | pBAD30XS with PEPPR2 - 2.2 inserted between the xhoI and speI sites |
| pBAD30XS45 | pBAD30XS with PEPPR4 - 0.5 inserted between the xhoI and speI sites |
| pBAD30XS46 | pBAD30XS with PEPPR5 - 0.2 inserted between the xhoI and speI sites |
| pBAD30XS47 | pBAD30XS with DPPS (mnmG) - 1.0 inserted between the xhoI and speI sites |
| pBAD30XS48 | pBAD30XS with DPPT - 1.2 inserted between the xhoI and speI sites |
| pBAD30XS49 | pBAD30XS with QPPS - 1.0 inserted between the xhoI and speI sites |
| pBAD30XS50 | pBAD30XS with QPPT (cytR) - 1.2 inserted between the xhoI and speI sites |
| pBAD30XS51 | pBAD30XS with VPPS (mgtA) - 1.0 inserted between the xhoI and speI sites |
| pBAD30XS52 | pBAD30XS with VPPT - 1.2 inserted between the xhoI and speI sites |
| pBAD30XS53 | pBAD30XS with DPPS2 - 1.1 inserted between the xhoI and speI sites |
| pBAD30XS54 | pBAD30XS with DPPS3 - 0.7 inserted between the xhoI and speI sites |
| pBAD30XS55 | pBAD30XS with DPPS6 - 1.5 inserted between the xhoI and speI sites |
| pBAD30XS56 | pBAD30XS with APPQ (visC) inserted between the xhoI and speI sites |
| pBAD30XS57 | pBAD30XS with NPPQ (yjhB) inserted between the xhoI and speI sites |
| pBAD30XS58 | pBAD30XS with TPPQ (yaaA) inserted between the xhoI and speI sites |
| pBAD30XS59 | pBAD30XS with SPPQ inserted between the xhoI and speI sites |
| pBAD30XS60 | pBAD30XS with RPPP (ytfM) inserted between the xhoI and speI sites |
| pBAD30XS61 | pBAD30XS with KPPP inserted between the xhoI and speI sites |
| pBAD30XS62 | pBAD30XS with IPPP (lepA) inserted between the xhoI and speI sites |
| pBAD30XS63 | pBAD30XS with LPPP (yeiG) inserted between the xhoI and speI sites |
| pBAD30XS64 | pBAD30XS with VPPP (ycgR) inserted between the xhoI and speI sites |
| pBAD30XS65 | pBAD30XS with PPPP (ycgL) inserted between the xhoI and speI sites |
| pBAD30XS66 | pBAD30XS with MLVVE (dcuA) inserted between the xhoI and speI sites |
| pBAD30XS67 | pBAD30XS with SAAWLG (nanT) inserted between the xhoI and speI sites |
| pBAD30XS68 | pBAD30XS with WEKQGYFKPNG (valS) inserted between the xhoI and speI sites |
| pBAD30XS69 | pBAD30XS with WFIPN (ycbZ) inserted between the xhoI and speI sites |
| pBAD30XS70 | pBAD30XS with MLDSIGPAA (gatC) inserted between the xhoI and speI sites |
| pBAD30XS71 | pBAD30XS with MGLDPGLRTG (yhgf) inserted between the xhoI and speI sites |
| pBAD30XS72 | pBAD30XS with STAKLKAAP (ampH) inserted between the xhoI and speI sites |
| pBAD30XS80 | pBAD30XS with TPPN inserted between the xhoI and speI sites |
| pBAD30XS81 | pBAD30XS with SPPN inserted between the xhoI and speI sites |
| pBAD30XSD1 | pBAD30XSD1 with PNPPK1a inserted between the xhoI and speI sites |
| pBAD30XSD2 | pBAD30XSD2 with PNPPK2a inserted between the xhoI and speI sites |
| pBAD30XSD3 | pBAD30XSD3 with PNPPK3a inserted between the xhoI and speI sites |
| pBAD30XSD4 | pBAD30XSD4 with PNPPK4a inserted between the xhoI and speI sites |
| pBAD30XSD5 | pBAD30XSD5 with PNPPK1b inserted between the xhoI and speI sites |
| pBAD30XSD6 | pBAD30XSD6 with PNPPK2b inserted between the xhoI and speI sites |
| pBAD30XSD7 | pBAD30XSD7 with PNPPK3b inserted between the xhoI and speI sites |
| pBAD30XSD8 | pBAD30XSD8 with PNPPK4b inserted between the xhoI and speI sites |
| pBAD30XSD9 | pBAD30XSD9 with PEPPK1a inserted between the xhoI and speI sites |
| pBAD30XSD10 | pBAD30XSD10 with PEPPK2a inserted between the xhoI and speI sites |
| pBAD30XSD11 | pBAD30XSD11 with PEPPK3a inserted between the xhoI and speI sites |
| pBAD30XSD12 | pBAD30XSD12 with PEPPK4a inserted between the xhoI and speI sites |
| pBAD30XSD13 | pBAD30XSD13 with PEPPK1b inserted between the xhoI and speI sites |
| pBAD30XSD14 | pBAD30XSD14 with PEPPK2b inserted between the xhoI and speI sites |
| pBAD30XSD15 | pBAD30XSD15 with PEPPK3b inserted between the xhoI and speI sites |
| pBAD30XSD16 | pBAD30XSD16 with PEPPK4b inserted between the xhoI and speI sites |
| pBAD30XSD17 | pBAD30XSD17 with PQPPK1a inserted between the xhoI and speI sites |
| pBAD30XSD18 | pBAD30XSD18 with PQPPK2a inserted between the xhoI and speI sites |
| pBAD30XSD19 | pBAD30XSD19 with PQPPK3a inserted between the xhoI and speI sites |
| pBAD30XSD20 | pBAD30XSD20 with PQPPK4a inserted between the xhoI and speI sites |
| pBAD30XSD21 | pBAD30XSD21 with PQPPK1b inserted between the xhoI and speI sites |
| pBAD30XSD22 | pBAD30XSD22 with PQPPK2b inserted between the xhoI and speI sites |
| pBAD30XSD23 | pBAD30XSD23 with PQPPK3b inserted between the xhoI and speI sites |
| pBAD30XSD24 | pBAD30XSD24 with PQPPK4b inserted between the xhoI and speI sites |
| pBAD30XSD25 | pBAD30XSD25 with PDPPK1a inserted between the xhoI and speI sites |
| pBAD30XSD26 | pBAD30XSD26 with PDPPK2a inserted between the xhoI and speI sites |
| pBAD30XSD27 | pBAD30XSD27 with PDPPK3a inserted between the xhoI and speI sites |
| pBAD30XSD28 | pBAD30XSD28 with PDPPK4a inserted between the xhoI and speI sites |
| pBAD30XSD29 | pBAD30XSD29 with PDPPK1b inserted between the xhoI and speI sites |
| pBAD30XSD30 | pBAD30XSD30 with PDPPK2b inserted between the xhoI and speI sites |
| pBAD30XSD31 | pBAD30XSD31 with PDPPK3b inserted between the xhoI and speI sites |
| pBAD30XSD32 | pBAD30XSD32 with PDPPK4b inserted between the xhoI and speI sites |
| pBAD30XSER1 | pBAD30XSR1 with PNPPS1-1.1 inserted between the xhoI and speI sites |
| pBAD30XSER2 | pBAD30XSR2 with PNPPS2-1.0 inserted between the xhoI and speI sites |
| pBAD30XSER3 | pBAD30XSR3 with PNPPS3-0.7 inserted between the xhoI and speI sites |
| pBAD30XSER5 | pBAD30XSR5 with PNPPS5-0.7 inserted between the xhoI and speI sites |
| pXG10sf | A low copy-number expression vector that places cloned genes under the control of the constitutively active tet0-1 promoter and in frame with ‘super-folder’ GFP [Corcoran, 2012 #77] |
| pXG10sf-LacZ | First 186 codons of *lacZ* in pXG10sf [Corcoran, 2012 #77] |
| pXG10sf-AtpD | pXG10sf with full-length *atpD* (excluding stop codon) inserted between NsiI and NheI sites. 74bp upstream of the start codon was included as a 5’ UTR |
| pXG10sf-AtpA | pXG10sf with full-length *atpA* (excluding stop codon) inserted between NsiI and NheI sites. 75bp upstream of the start codon was included as a 5’ UTR |
| pXG10sf-AtpD P214L | pXG10sf-AtpD with P214L mutation |
| pXG10sf-AtpA P281L | pXG10sf-AtpA with P281L mutation |
| pXG10sf-AtpA R279P | pXG10sf-AtpA with R279P mutation |
| pXG10sf-AtpD 40aa swap | pXG10sf-AtpD with 40 codons upstream of the PPG motif replaced by those from *atpA* |
| pXG10sf-AtpD 24aa swap | pXG10sf-AtpD with 24 codons upstream of the PPG motif replaced by those from *atpA* |
| pXG10sf-AtpD 12aa swap | pXG10sf-AtpD with 12 codons upstream of the PPG motif replaced by those from *atpA* |
| pXG10sf-AtpD 6aa swap | pXG10sf-AtpD with 6 codons upstream of the PPG motif replaced by those from *atpA* |
| pXG10sf-AtpD 4aa swap | pXG10sf-AtpD with 4 codons upstream of the PPG motif replaced by those from *atpA* |
| pXG10sf-AtpD 2aa swap | pXG10sf-AtpD with 2 codons upstream of the PPG motif replaced by those from *atpA* |
| pXG10sf-AtpD E212R | pXG10sf-AtpD with E212R mutation |
| pXG10sf-AtpD N211R | pXG10sf-AtpD with N211R mutation |
| pXG10sf-AtpD M210L | pXG10sf-AtpD with M210L mutation |
| pXG10sf-AtpA 40aa swap | pXG10sf-AtpA with 40 codons upstream of the PPG motif replaced by those from *atpD* |
| pXG10sf-AtpA 24aa swap | pXG10sf-AtpA with 24 codons upstream of the PPG motif replaced by those from *atpD* |
| pXG10sf-AtpA 12aa swap | pXG10sf-AtpA with 12 codons upstream of the PPG motif replaced by those from *atpD* |
| pXG10sf-AtpA 6aa swap | pXG10sf-AtpA with 6 codons upstream of the PPG motif replaced by those from *atpD* |
| pXG10sf-AtpA 4aa swap | pXG10sf-AtpA with 4 codons upstream of the PPG motif replaced by those from *atpD* |
| pXG10sf-AtpA 2aa swap | pXG10sf-AtpA with 2 codons upstream of the PPG motif replaced by those from *atpD* |
| pXG10sf-AtpA R279E | pXG10sf-AtpA with R279E mutation |
| pXG10sf-AtpA R278N | pXG10sf-AtpA with R278N mutation |
| pXG10sf-AtpA L277M | pXG10sf-AtpA with L277M mutation |
| pXG10sf-AtpD QM209-10LL | pXG10sf-AtpD with Q209L and M210L mutations |
| pXG10sf-AtpD YG207-8SL | pXG10sf-AtpD with Y207S and G208L mutations |
| pXG10sf-AtpD LV205-6QI | pXG10sf-AtpD with L205Q and V206I mutations |
| pXG10sf-AtpD VS203-4YR | pXG10sf-AtpD with V203Y and S204R mutations |
| pXG10sf-AtpD DK201-2VA | pXG10sf-AtpD with D201V and K202A mutations |
| pXG10sf-AtpD Q209L | pXG10sf-AtpD with Q209L mutation |
| pXG10sf-AtpD G208L | pXG10sf-AtpD with G208L mutation |
| pXG10sf-AtpD Y207S | pXG10sf-AtpD with Y207S mutation |
| pXG10sf-AtpA LL276-7QM | pXG10sf-AtpA with L276Q and L277M mutations |
| pXG10sf-AtpA SL274-5YG | pXG10sf-AtpA with S274Y and L275G mutations |
| pXG10sf-AtpA QI272-3LV | pXG10sf-AtpA with Q272L and I273V mutations |
| pXG10sf-AtpA YR270-1VS | pXG10sf-AtpA with Y270V and R271S mutations |
| pXG10sf-AtpA VA268-9DK | pXG10sf-AtpA with V268D and A269K mutations |
| pXG10sf-AtpA L276Q | pXG10sf-AtpA with L276Q mutation |
| pXG10sf-AtpA L275G | pXG10sf-AtpA with L275G mutation |
| pXG10sf-AtpA S274Y | pXG10sf-AtpA with S274Y mutation |
| pXG10sf-AtpD P214 CCG::CCA | pXG10sf-AtpD with P214 codon mutated from CCG to CCA |
| pXG10sf-AtpD E212 GAG::GAA | pXG10sf-AtpD with E212 codon mutated from GAG to GAA |
| pXG10sf-AtpA R278 GCT::AGG | pXG10sf-AtpD with R278 codon mutated from GCT to AGG |
| pXG10sf-AtpA R279 CGT::AGG | pXG10sf-AtpD with R279 codon mutated from GCT to AGG |
| pXG10sf-AtpA R278-9 CGTCGT ::AGGAGG | pXG10sf-AtpD with both R278 and R279 codons mutated from GCT to AGG |

**Primers**

| Purpose | Name | Sequence (5’ to 3’) |
| --- | --- | --- |
| pBAD30X701 | XSF | CTAGCGAATTCAGGAGGAATTTACCATGAGTAAACTCGAGGGCAACACTAGTAGAGAAGAACTTTTCACTG |
|  | MR_70 | GACTCTAGAGGATCCCCGGGTACCC |
| pBAD30X702 | X2F | TCGAGCCGCCGCCGCCGCCGCCGA |
|  | X2R | CTAGTCGGCGGCGGCGGCGGCGGC |
| pBAD30X703 | X3F | TCGAGCCGCCGGGTA |
|  | X3R | CTAGTACCCGGCGGC |
| pBAD30X704 | X4F | TCGAGCCGCCGAACA |
|  | X4R | CTAGTGTTCGGCGGC |
| pBAD30X705 | X5F | TCGAGGGGCAGATCGCCGCCGCACTGGCGGCAA |
|  | X5R | CTAGTTGCCGCCAGTGCGGCGGCGATCTGCCCC |
| pBAD30X725 | X25F | TCGAGAACCCGCCGAACA |
|  | X25R | CTAGTGTTCGGCGGGTTC |
| pBAD30X727 | X27F | TCGAGCCGCCAAAAA |
|  | X27R | CTAGTTTTTGGCGGC |
| pBAD30X728 | X28F | TCGAGCTCCCACCGCCAGCGAAGA |
|  | X28R | CTAGTCTTCGCTGGCGGTGGGAGC |
| pBAD30X729 | X29F | TCGAGCCGGAGCCGCCAAAGA |
|  | X29R | CTAGTCTTTGGCGGCTCCGGC |
| pBAD30X730 | X30F | TCGAGCCGGAGCCGCCACGTA |
|  | X30R | CTAGTACGTGGCGGCTCCGGC |
| pBAD30X731 | X31F | TCGAGCCGGAGCCGCCACATA |
|  | X31R | CTAGTATGTGGCGGCTCCGGC |
| pBAD30X732 | X32F | TCGAGCCGAAACCGCCAAAAA |
|  | X32R | CTAGTTTTTGGCGGTTTCGGC |
| pBAD30X733 | X33F | TCGAGCCGGTTCCGCCAAAAA |
|  | X33R | CTAGTTTTTGGCGGAACCGGC |
| pBAD30X734 | X34F | TCGAGCCGACCCCGCCAAAAA |
|  | X34R | CTAGTTTTTGGCGGGGTCGGC |
| pBAD30X735 | X35F | TCGAGCCGTTTCCGCCAAAAA |
|  | X35R | CTAGTTTTTGGCGGAAACGGC |
| pBAD30X736 | X36F | TCGAGCTGCCGCCGCTGA |
|  | X36R | CTAGTCAGCGGCGGCAGC |
| pBAD30X737 | X37F | TCGAGCTGCCACCGCGTA |
|  | X37R | CTAGTACGCGGTGGCAGC |
| pBAD30X738 | X38F | TCGAGAAACCACCGAAGA |
|  | X38R | CTAGTCTTCGGTGGTTTC |
| pBAD30X739 | X39F | TCGAGGTGCCACCAGATA |
|  | X39R | CTAGTATCTGGTGGCACC |
| pBAD30X740 | X40F | TCGAGATTCCGCCAAATA |
|  | X40R | CTAGTATTTGGCGGAATC |
| pBAD30X741 | X41F | TCGAGCATCCACCGGAGA |
|  | X41R | CTAGTCTCCGGTGGATGC |
| pBAD30X742 | X42F | TCGAGGCGCCACCTAACA |
|  | X42R | CTAGTGTTAGGTGGCGCC |
| pBAD30X743 | X43F | TCGAGCGTCCACCGCCAAAGA |
|  | X43R | CTAGTCTTTGGCGGTGGACGC |
| pBAD30X744 | X44F | TCGAGCCGGAGCCGCCACGCA |
|  | X44R | CTAGTGCGTGGCGGCTCCGGC |
| pBAD30X745 | X45F | TCGAGCCGGAGCCGCCACGGA |
|  | X45R | CTAGTCCGTGGCGGCTCCGGC |
| pBAD30X746 | X46F | TCGAGCCGGAGCCGCCAAGAA |
|  | X46R | CTAGTTCTTGGCGGCTCCGGC |
| pBAD30X747 | X47F | TCGAGGATCCGCCGTCCA |
|  | X47R | CTAGTGGACGGCGGATCC |
| pBAD30X748 | X48F | TCGAGGATCCGCCGACTA |
|  | X48R | CTAGTAGTCGGCGGATCC |
| pBAD30X749 | X49F | TCGAGCAACCGCCTTCCA |
|  | X49R | CTAGTGGAAGGCGGTTGC |
| pBAD30X750 | X50F | TCGAGCAACCGCCTACTA |
|  | X50R | CTAGTAGTAGGCGGTTGC |
| pBAD30X751 | X51F | TCGAGGTCCCGCCGTCCA |
|  | X51R | CTAGTGGACGGCGGGACC |
| pBAD30X752 | X52F | TCGAGGTCCCGCCGACTA |
|  | X52R | CTAGTAGTCGGCGGGACC |
| pBAD30X753 | X53F | TCGAGGATCCGCCGTCTA |
|  | X53R | CTAGTAGACGGCGGATCC |
| pBAD30X754 | X54F | TCGAGGATCCGCCGTCAA |
|  | X54R | CTAGTTGACGGCGGATCC |
| pBAD30X755 | X55F | TCGAGGATCCGCCGAGCA |
|  | X55R | CTAGTGCTCGGCGGATCC |
| pBAD30X756 | X56F | TCGAGGCACCACCACAAA |
|  | X56R | CTAGTTTGTGGTGGTGCC |
| pBAD30X757 | X57F | TCGAGAATCCACCACAAA |
|  | X57R | CTAGTTTGTGGTGGATTC |
| pBAD30X758 | X58F | TCGAGACGCCTCCGCAGA |
|  | X58R | CTAGTCTGCGGAGGCGTC |
| pBAD30X759 | X59F | TCGAGTCGCCTCCGCAGA |
|  | X59R | CTAGTCTGCGGAGGCGAC |
| pBAD30X760 | X60F | TCGAGCGTCCACCGCCAA |
|  | X60R | CTAGTTGGCGGTGGACGC |
| pBAD30X761 | X61F | TCGAGAAACCACCGCCAA |
|  | X61R | CTAGTTGGCGGTGGTTTC |
| pBAD30X762 | X62F | TCGAGATTCCGCCGCCGA |
|  | X62R | CTAGTCGGCGGCGGAATC |
| pBAD30X763 | X63F | TCGAGCTCCCTCCACCTA |
|  | X63R | CTAGTAGGTGGAGGGAGC |
| pBAD30X764 | X64F | TCGAGGTACCGCCTCCCA |
|  | X64R | CTAGTGGGAGGCGGTACC |
| pBAD30X765 | X65F | TCGAGTTACCGCCACCAA |
|  | X65R | CTAGTTGGTGGCGGTAAC |
| pBAD30X766 | X66F | TCGAGATGCTAGTTGTAGAAA |
|  | X66R | CTAGTTTCTACAACTAGCATC |
| pBAD30X767 | X67F | TCGAGTCCGCTGCCTGGTTGGGAA |
|  | X67R | CTAGTTCCCAACCAGGCAGCGGAC |
| pBAD30X768 | X68F | TCGAGTGGGAAAAGCAGGGCTACTTTAAGCCTAATGGCA |
|  | X68R | CTAGTGCCATTAGGCTTAAAGTAGCCCTGCTTTTCCCAC |
| pBAD30X769 | X69F | TCGAGTGGTTTATTCCGAACA |
|  | X69R | CTAGTGTTCGGAATAAACCAC |
| pBAD30X770 | X70F | TCGAGATGCTGGATTCCATTGGCCCGGCGGCGA |
|  | X70R | CTAGTCGCCGCCGGGCCAATGGAATCCAGCATC |
| pBAD30X771 | X71F | TCGAGATGGGCCTCGATCCGGGTCTGCGTACTGGGA |
|  | X71R | CTAGTCCCAGTACGCAGACCCGGATCGAGGCCCATC |
| pBAD30X772 | X72F | TCGAGTCTACGGCGAAGCTGAAAGCCGCGCCAA |
|  | X72R | CTAGTTGGCGCGGCTTTCAGCTTCGCCGTAGAC |
| pBAD30X780 | X80F | TCGAGACGCCTCCGAACA |
|  | X80R | CTAGTGTTCGGAGGCGTC |
| pBAD30X781 | X81F | TCGAGTCGCCTCCGAACA |
|  | X81R | CTAGTGTTCGGAGGCGAC |
| pBAD30XSD1 | SD1F | TCGAGCCGAACCCGCCAAAAA |
|  | SD1R | CTAGTTTTTGGCGGGTTCGGC |
| pBAD30XSD2 | SD2F | TCGAGCCCAACCCGCCAAAAA |
|  | SD2R | CTAGTTTTTGGCGGGTTGGGC |
| pBAD30XSD3 | SD3F | TCGAGCCAAACCCGCCAAAAA |
|  | SD3R | CTAGTTTTTGGCGGGTTTGGC |
| pBAD30XSD4 | SD4F | TCGAG CCTAATCCGCCAAAAA |
|  | SD4R | CTAGTTTTTGGCGGATTAGGC |
| pBAD30XSD5 | SD5F | TCGAGCCGAATCCGCCAAAAA |
|  | SD5R | CTAGTTTTTGGCGGATTCGGC |
| pBAD30XSD6 | SD6 F | TCGAGCCCAATCCGCCAAAAA |
|  | SD6 R | CTAGTTTTTGGCGGATTGGGC |
| pBAD30XSD7 | SD7 F | TCGAGCCAAATCCGCCAAAAA |
|  | SD7 R | CTAGTTTTTGGCGGATTTGGC |
| pBAD30XSD8 | SD8F | TCGAGCCTAATCCGCCAAAAA |
|  | SD8R | CTAGTTTTTGGCGGATTAGGC |
| pBAD30XSD9 | SD9F | TCGAG CCGGAGCCGCCAAAA A |
|  | SD9R | CTAGTTTTTGGCGGCTCCGGC |
| pBAD30XSD10 | SD10 F | TCGAGCCCGAGCCGCCAAAAA |
|  | SD10 R | CTAGTTTTTGGCGGCTCGGGC |
| pBAD30XSD11 | SD11F | TCGAGCCAGAGCCGCCAAAAA |
|  | SD11R | CTAGTTTTTGGCGGCTCTGGC |
| pBAD30XSD12 | SD12F | TCGAGCCTGAACCGCCAAAAA |
|  | SD12R | CTAGTTTTTGGCGGTTCAGGC |
| pBAD30XSD13 | SD13F | TCGAGCCGGAACCGCCAAAAA |
|  | SD13R | CTAGTTTTTGGCGGTTCCGGC |
| pBAD30XSD14 | SD14F | TCGAGCCCGAACCGCCAAAAA |
|  | SD14R | CTAGTTTTTGGCGGTTCGGGC |
| pBAD30XSD15 | SD15F | TCGAGCCAGAACCGCCAAAAA |
|  | SD15R | CTAGTTTTTGGCGGTTCTGGC |
| pBAD30XSD16 | SD16F | TCGAGCCTGAACCGCCAAAAA |
|  | SD16R | CTAGTTTTTGGCGGTTCAGGC |
| pBAD30XSD17 | SD17F | TCGAGCCGCAACCGCCAAAAA |
|  | SD17R | CTAGTTTTTGGCGGTTGCGGC |
| pBAD30XSD18 | SD18F | TCGAGCCCCAACCGCCAAAAA |
|  | SD18R | CTAGTTTTTGGCGGTTGGGGC |
| pBAD30XSD19 | SD19F | TCGAGCCACAACCGCCAAAAA |
|  | SD19R | CTAGTTTTTGGCGGTTGTGGC |
| pBAD30XSD20 | SD20F | TCGAGCCTCAACCGCCAAAAA |
|  | SD20R | CTAGTTTTTGGCGGTTGAGGC |
| pBAD30XSD21 | SD21F | TCGAGCCGCAGCCGCCAAAAA |
|  | SD21R | CTAGTTTTTGGCGGCTGCGGC |
| pBAD30XSD22 | SD22F | TCGAGCCCCAGCCGCCAAAAA |
|  | SD22R | CTAGTTTTTGGCGGCTGGGGC |
| pBAD30XSD23 | SD23F | TCGAGCCACAGCCGCCAAAAA |
|  | SD23R | CTAGTTTTTGGCGGCTGTGGC |
| pBAD30XSD24 | SD24F | TCGAGCCTCAGCCGCCAAAAA |
|  | SD24R | CTAGTTTTTGGCGGCTGAGGC |
| pBAD30XSD25 | SD25F | TCGAGCCGGATCCGCCAAAAA |
|  | SD25R | CTAGTTTTTGGCGGATCCGGC |
| pBAD30XSD26 | SD26F | TCGAGCCCGATCCGCCAAAAA |
|  | SD26R | CTAGTTTTTGGCGGATCGGGC |
| pBAD30XSD27 | SD27F | TCGAGCCAGATCCGCCAAAAA |
|  | SD27R | CTAGTTTTTGGCGGATCTGGC |
| pBAD30XSD28 | SD28F | TCGAGCCTGATCCGCCAAAAA |
|  | SD28R | CTAGTTTTTGGCGGATCAGGC |
| pBAD30XSD29 | SD29F | TCGAGCCGGACCCGCCAAAAA |
|  | SD29R | CTAGTTTTTGGCGGGTCCGGC |
| pBAD30XSD30 | SD30F | TCGAGCCCGACCCGCCAAAAA |
|  | SD30R | CTAGTTTTTGGCGGGTCGGGC |
| pBAD30XSD31 | SD31F | TCGAGCCAGACCCGCCAAAAA |
|  | SD31R | CTAGTTTTTGGCGGGTCTGGC |
| pBAD30XSD32 | SD32F | TCGAGCCTGACCCGCCAAAAA |
|  | SD32R | CTAGTTTTTGGCGGGTCAGGC |
| pBAD30XSR1 | SR1F | TCGAGCCGAACCCGCCATCTA |
|  | SR1R | CTAGTAGATGGCGGGTTCGGC |
| pBAD30XSR2 | SR2F | TCGAGCCGAACCCGCCATCCA |
|  | SR2R | CTAGTGGATGGCGGGTTCGGC |
| pBAD30XSR3 | SR3F | TCGAGCCGAACCCGCCATCAA |
|  | SR3R | CTAGTTGATGGCGGGTTCGGC |
| pBAD30XSR5 | SR5F | TCGAGCCGAACCCGCCAAGTA |
|  | SR5R | CTAGTACTTGGCGGGTTCGGC |
| pBAD30XSR6 | SR6F | TCGAGCCGAACCCGCCAAGCA |
|  | SR6R | CTAGTGCTTGGCGGGTTCGGC |
| pXG10sf-AtpD | AtpD F NsiI | GTTTTTATGCATCATTACTCAGGAACTCACCGAG |
|  | AtpD FL R NheI | GTTTTTGCTAGCAAGTTTTTTGGCTTTTTCCACGG |
| pXG10sf-AtpA | AtpA F NsiI | GTTTTTATGCATATGGTCATTGATGGCAGCG |
|  | AtpA FL R NheI | GTTTTTGCTAGCCCAGGACTGGGTTGCTTTG |
| pXG10sf-AtpD P214L | atpD P214L SDM F & R | TGGCCAGATGAACGAGCCGCTGGGAAACCGTCTGCGCGTTG |
|  |  | CAACGCGCAGACGGTTTCCCAGCGGCTCGTTCATCTGGCCA |
| pXG10sf-AtpA P281L | atpA P281L SDM ext F & R | CCTGCTGCTCCGTCGTCCGCTGGGACGTGAAGCATTCCCGGGCGACGTATTCTACCTCC |
|  |  | CCCGGGAATGCTTCACGTCCCAGCGGACGACGGAGCAGCAGGGAGATCTGACGGTAAGC |
| pXG10sf-AtpA R279P | AtpA R279P SDM F & R | CGTCAGATCTCCCTGCTGCTCCGTCCTCCGCCAGGACGTGAAGCATTCCCG |
|  |  | CGGGAATGCTTCACGTCCTGGCGGAGGACGGAGCAGCAGGGAGATCTGACG |
| pXG10sf-AtpD 40aa swap | atpD- atpA 40aa Uflank R | GTATTCGCCCATTGCGCAACCGGCATAGGAGTGCTCGATCGCGATGTTACGGATAAGCTC |
|  | atpA 40aa Uflank ins F & R | TATGCCGGTTGCGCAATGGGCGAATACTTC |
|  |  | ACGACGGAGCAGCAGGGAGATCTGAC |
| pXG10sf-AtpD 24aa swap | atpD atpA 24aa Uflank F & R | TGATCATTTACGATGACCTGTCTAAACAGGCTGTTGCTTACCGTCAGATCTCCCTGC |
|  |  | TGTTTAGACAGGTCATCGTAAATGATCAGCGCGTTACCCTCACGAGTACGTTCCCCT |
| pXG10sf-AtpD 12aa swap | New atpD-atpA 12aa Uflank F & R | TTACCGTCAGATCTCCCTGCTGCTCCGTCGTCCGCCGGGAAACCGTCTGCGCGTTGCATT |
|  |  | GGAGCAGCAGGGAGATCTGACGGTAAGCAACGATAACGTTGGAGTCGGTCATTTCGTGGT |
| pXG10sf-AtpD 6aa swap | atpD-A 6aa Uflank F & R | GTGTCCCTGCTGCTCCGTCGTCCGCCGGGAAACCGTCTGCGCGTTGCATTGACC |
|  |  | CGGACGACGGAGCAGCAGGGACACCAGGGATACTTTATCGATAACGTTGGAGTCGGTC |
| pXG10sf-AtpD 4aa swap | atpD-A 4aa Uflank F & R | GTGTATGGCCTGCTCCGTCGTCCGCCGGGAAACCGTCTGCGCGTTGCATTGACC |
|  |  | CGGACGACGGAGCAGGCCATACACCAGGGATACTTTATCGATAACGTTGGAGTCGGTC |
| pXG10sf-AtpD 2aa swap | atpD NE211-2RR F & R | GTGTATGGCCAGATGCGTCGTCCGCCGGGAAACCGTCTGCGCGTTGCATTGACC |
|  |  | CGGACGACGCATCTGGCCATACACCAGGGATACTTTATCGATAACGTTGGAGTCGG |
| pXG10sf-AtpD E212R | atpD E212R F & R | GTGTATGGCCAGATGAACCGTCCGCCGGGAAACCGTCTGCGCGTTGCATTGACC |
|  |  | CGGACGGTTCATCTGGCCATACACCAGGGATACTTTATCGATAACGTTGGAGTCGG |
| pXG10sf-AtpD N211R | atpD N211R F & R | GTGTATGGCCAGATGCGTGAGCCGCCGGGAAACCGTCTGCGCGTTGCATTGACC |
|  |  | CGGCTCACGCATCTGGCCATACACCAGGGATACTTTATCGATAACGTTGGAGTCGG |
| pXG10sf-AtpD M210L | atpD M210L F & R | GTGTATGGCCAGCTCAACGAGCCGCCGGGAAACCGTCTGCGCGTTGCATTGACC |
|  |  | CGGCTCGTTGAGCTGGCCATACACCAGGGATACTTTATCGATAACGTTGGAGTCGG |
| pXG10sf-AtpA 40aa swap | atpA atpD 40aa Uflank R | TTCCCCTACGCCCGCAAACACTGAGTAACCCGGCGCCAGATATTGCAGCGC |
|  | atpD 40aa Uflank ins F & R | GGTTACTCAGTGTTTGCGGGC |
|  |  | CTCGTTCATCTGGCCATACACC |
| pXG10sf-AtpA 24aa swap | atpA atpD 24aa Uflank F & R | TACCACGAAATGACCGACTCCAACGTTATCGATAAAGTATCCCTGGTGTATGGCCAGATG |
|  |  | TAACGTTGGAGTCGGTCATTTCGTGGTAGAAGTCATCTTCGCCGCGGTCACGGAAG |
| pXG10sf-AtpA 12aa swap | atpA-atpD 12aa Uflank F & R | GATAAAGTATCCCTGGTGTATGGCCAGATGAACGAGCCGCCAGGACGTGAAGCA |
|  |  | CTCGTTCATCTGGCCATACACCAGGGATACTTTATCAGCCTGTTTAGACAGGTCATCGTA |
| pXG10sf-AtpA 6aa swap | atpA-D 6aa Uflank F & R | CAGATCTATGGCCAGATGAACGAGCCGCCAGGACGTGAAGCATTCCCGGGC |
|  |  | CTCGTTCATCTGGCCATAGATCTGACGGTAAGCAACAGCCTGTTTAGACAGGTCATCG |
| pXG10sf-AtpA 4aa swap | atpA-D 4aa Uflank F & R | CAGATCTCCCTGCAGATGAACGAGCCGCCAGGACGTGAAGCATTCCCGGGC |
|  |  | CTCGTTCATCTGCAGGGAGATCTGACGGTAAGCAACAGCCTGTTTAGACAGGTCATCG |
| pXG10sf-AtpA 2aa swap | atpA RR278-9NE F & R | CAGATCTCCCTGCTGCTCAACGAGCCGCCAGGACGTGAAGCATTCCCGGGC |
|  |  | CTCGTTGAGCAGCAGGGAGATCTGACGGTAAGCAACAGCCTGTTTAGACAGGTCATCG |
| pXG10sf-AtpA R279E | atpA R279E F & R | CAGATCTCCCTGCTGCTCCGTGAGCCGCCAGGACGTGAAGCATTCCCGGGC (51) |
|  |  | CTCACGGAGCAGCAGGGAGATCTGACGGTAAGCAACAGCCTGTTTAGACAGGTCATCG (58) |
| pXG10sf-AtpA R278N | atpA R278N F & R | CAGATCTCCCTGCTGCTCAACCGTCCGCCAGGACGTGAAGCATTCCCGGGC (51) |
|  |  | ACGGTTGAGCAGCAGGGAGATCTGACGGTAAGCAACAGCCTGTTTAGACAGGTCATCG (58) |
| pXG10sf-AtpA L277M | atpA L277M F & R | CAGATCTCCCTGCTGATGCGTCGTCCGCCAGGACGTGAAGCATTCCCGGGC (51) |
|  |  | ACGACGCATCAGCAGGGAGATCTGACGGTAAGCAACAGCCTGTTTAGACAGGTCATCG (58) |
| pXG10sf-AtpD QM209-10LL | atpD QM209-10LL F & R | GTGTATGGCCTGCTCAACGAGCCGCCGGGAAACCGTCTGCGCGTTGCATTGACC |
|  |  | CGGCTCGTTGAGCAGGCCATACACCAGGGATACTTTATCGATAACGTTGGAGTCGG |
| pXG10sf-AtpD YG207-8SL | atpD YG207-8SL F & R | GTGTCCCTGCAGATGAACGAGCCGCCGGGAAACCGTCTGCGCGTTGCATTGACC |
|  |  | CGGCTCGTTCATCTGCAGGGACACCAGGGATACTTTATCGATAACGTTGGAGTCGGTC |
| pXG10sf-AtpD LV205-6QI | atpD LV205-6QI F & R | GTTATCGATAAAGTATCCCAGATCTATGGCCAGATGAACGAGCCGCCGGGAAACCG |
|  |  | GATCTGGGATACTTTATCGATAACGTTGGAGTCGGTCATTTCGTGGTAGAAGTCGTTACC |
| pXG10sf-AtpD VS203-4YR | atpD VS203-4YR F & R | GTTATCGATAAATACCGTCTGGTGTATGGCCAGATGAACGAGCCGCCGGGAAACCG |
|  |  | CACCAGACGGTATTTATCGATAACGTTGGAGTCGGTCATTTCGTGGTAGAAGTCGTTACC |
| pXG10sf-AtpD DK201-2VA | atpD DK201-2VA F & R | GTTATCGTTGCTGTATCCCTGGTGTATGGCCAGATGAACGAGCCGCCGGGAAACCG |
|  |  | CACCAGGGATACAGCAACGATAACGTTGGAGTCGGTCATTTCGTGGTAGAAGTCGTTACC |
| pXG10sf-AtpD Q209L | atpD Q209L F & R | GTGTATGGCCTGATGAACGAGCCGCCGGGAAACCGTCTGCGCGTTGCATTGACC |
|  |  | CGGCTCGTTCATCAGGCCATACACCAGGGATACTTTATCGATAACGTTGGAGTCGG |
| pXG10sf-AtpD G208L | atpD G208L F & R | GTGTATCTGCAGATGAACGAGCCGCCGGGAAACCGTCTGCGCGTTGCATTGACC |
|  |  | CGGCTCGTTCATCTGCAGATACACCAGGGATACTTTATCGATAACGTTGGAGTCGG |
| pXG10sf-AtpD Y207S | atpD Y207S F & R | GTGTCCGGCCAGATGAACGAGCCGCCGGGAAACCGTCTGCGCGTTGCATTGACC |
|  |  | CGGCTCGTTCATCTGGCCGGACACCAGGGATACTTTATCGATAACGTTGGAGTCGGTC |
| pXG10sf-AtpA LL276-7QM | atpA LL276-7QM F & R | CAGATCTCCCTGCAGATGCGTCGTCCGCCAGGACGTGAAGCATTCCCGGGC |
|  |  | ACGACGCATCTGCAGGGAGATCTGACGGTAAGCAACAGCCTGTTTAGACAGGTCATCG |
| pXG10sf-AtpA SL274-5YG | atpA SL274-5YG F & R | CAGATCTATGGCCTGCTCCGTCGTCCGCCAGGACGTGAAGCATTCCCGGGC |
|  |  | ACGACGGAGCAGGCCATAGATCTGACGGTAAGCAACAGCCTGTTTAGACAGGTCATCG |
| pXG10sf-AtpA QI272-3LV | atpA QI272-3LV F & R | CAGGCTGTTGCTTACCGTCTGGTGTCCCTGCTGCTCCGTCGTCCGCCAGG |
|  |  | CACCAGACGGTAAGCAACAGCCTGTTTAGACAGGTCATCGTAAATGATCAGCGCATC |
| pXG10sf-AtpA YR270-1VS | atpA YR270-1VS F & R | CAGGCTGTTGCTGTATCCCAGATCTCCCTGCTGCTCCGTCGTCCGCCAGG |
|  |  | GATCTGGGATACAGCAACAGCCTGTTTAGACAGGTCATCGTAAATGATCAGCGCATC |
| pXG10sf-AtpA VA268-9DK | atpA VA268-9DK F & R | CAGGCTGATAAATACCGTCAGATCTCCCTGCTGCTCCGTCGTCCGCCAGG |
|  |  | GATCTGACGGTATTTATCAGCCTGTTTAGACAGGTCATCGTAAATGATCAGCGCATC |
| pXG10sf-AtpA L276Q | atpA L276Q F & R | CAGATCTCCCTGCAGCTCCGTCGTCCGCCAGGACGTGAAGCATTCCCGGGC |
|  |  | ACGACGGAGCTGCAGGGAGATCTGACGGTAAGCAACAGCCTGTTTAGACAGGTCATCG |
| pXG10sf-AtpA L275G | atpA L275G F & R | CAGATCTCCGGCCTGCTCCGTCGTCCGCCAGGACGTGAAGCATTCCCGGGC |
|  |  | ACGACGGAGCAGGCCGGAGATCTGACGGTAAGCAACAGCCTGTTTAGACAGGTCATCG |
| pXG10sf-AtpA S274Y | atpA S274Y F & R | CAGATCTATCTGCTGCTCCGTCGTCCGCCAGGACGTGAAGCATTCCCGGGC |
|  |  | ACGACGGAGCAGCAGATAGATCTGACGGTAAGCAACAGCCTGTTTAGACAGGTCATCG |
| pXG10sf-AtpD P214 CCG::CCA | atpD P214 CCG::CCA SDM F & R | ATGGCCAGATGAACGAGCCGCCAGGAAACCGTCTGCGCGTTGCAT |
|  |  | ATGCAACGCGCAGACGGTTTCCTGGCGGCTCGTTCATCTGGCCAT |
| pXG10sf-AtpD E212 GAG::GAA | atpD E212-GAA F & R | CCAGATGAACGAACCGCCGGGAAACCGTCTGCGCGTTGCATTGACC |
|  |  | GTTTCCCGGCGGTTCGTTCATCTGGCCATACACCAGGGATACTTTATCG |
| pXG10sf-AtpA R278 GCT::AGG | atpA R278-AGG F & R | CCCTGCTGCTCAGGCGTCCGCCAGGACGTGAAGCATTCCCGGGCGACGTATTC |
|  |  | CCTGGCGGACGCCTGAGCAGCAGGGAGATCTGACGGTAAGCAACAGCCTGTTTAG |
| pXG10sf-AtpA R279 CGT::AGG | atpA R279-AGG F & R | CTGCTGCTCCGTAGGCCGCCAGGACGTGAAGCATTCCCGGGCGACGTATTCTACC |
|  |  | GTCCTGGCGGCCTACGGAGCAGCAGGGAGATCTGACGGTAAGCAACAGCCTG |
| pXG10sf-AtpA R278-9 CGTCGT ::AGGAGG | atpA RR278-9-AGGAGG F & R | CCTGCTGCTCAGGAGGCCGCCAGGACGTGAAGCATTCCCGGGCGACGTATTCTACC |
|  |  | GTCCTGGCGGCCTCCTGAGCAGCAGGGAGATCTGACGGTAAGCAACAGCCTGTTTAG |
