# Supplementary material for: EF-P Dependent Pauses Integrate Proximal and Distal Signals during Translation
Source: PLoS Genet. 2014 Aug 21;10(8):e1004553. doi: 10.1371/journal.pgen.1004553 (PMC4140641; doi:10.1371/journal.pgen.1004553)
Supplement: Text S1 — Additional experimental procedures. (DOCX) [file pgen.1004553.s017.docx]

**Text S1. Additional Experimental Procedures**

*Preparation of ribosome footprint samples*

As previously described [[1](#_ENREF_1)], the frozen lysate after thawing on ice was supplemented with 5 mM CaCl_2_. Digestion was carried out at 25ºC in a thermomixer (Eppendorf) shaken at 550 rpm with 750 U micrococcal nuclease (Roche, catalog no. 10107921001) for each 0.5 mg RNA. Digestions were quenched after 1 hr with EGTA to a final concentration of 6 mM. Both digested and control samples were loaded onto 10/45% sucrose gradients (buffered in 10 mM MgCl_2_, 100 mM NH_4_Cl, 20 mM Tris pH 8.0, 2 mM DTT). The gradients were separated by ultracentrifugation at 35,000 rpm with SW41 rotor for 2.5 hr at 4ºC. Fractions were harvested from the gradient and monosomes were manually collected by monitoring OD_254 nm_.

*mRNA fragmentation*

As previously described [[2](#_ENREF_2)], total RNA for each strain was prepared from the same lysate used for ribosome footprinting. Ribosomal RNA was removed from the total RNA with MICROBExpress (Ambion). Small RNA was removed with MEGAclear (Ambion). The mRNA was randomly fragmented by partial alkaline hydrolysis as previously described [[3](#_ENREF_3)].

*Library Generation*

Ribosome footprints and the fragmented mRNA samples were converted to a cDNA library as previously described [[1](#_ENREF_1),[3](#_ENREF_3)]. Samples were first denatured using SDS to a final concentration of 1% (w/v). Following that, the denatured samples were extracted once with 1 volume of hot acid phenol then once with 1 volume of acid phenol and finally with 1 volume of chloroform–IAA (24:1). The precipitated yield was resuspended in 20 µl of 10 mM Tris pH 7.0. To resolve, 25 mg of RNA was mixed with 2x TBE-urea sample loading buffer (Invitrogen) and loaded on a 15% TBE-urea gel. The gel was run in 1x TBE at 200V then stained for 3 min in SYBR Gold. With the 10 bp ladder (Invitrogen), a band between 28 to 42 bp was excised; gel purified, and resuspended in 10 mM Tris pH 7.0. Dephosphorylation of the 3 ́ ends of the RNA was done by adding T4 PNK (NEB) for 1 hr at 37ºC; followed by heat inactivation. The precipitated RNA was resuspended in 10 µl of 10 mM Tris pH 7.0 . From this, 5 pmol (quantified by Bio-Analyzer) of RNA was diluted to 5 µl in 10 mM Tris pH 7.0 and ligated for 2.5 hr at 37ºC to 1 µl of 1 µg/µl Linker-1 (5 **́**_App/CTGTAGGCACCATCAAT/3 ddC_ **3 ́**, IDTDNA) with 8 µl of 50% sterile filtered PEG MW 8000, 2 µl of 100% DMSO (Sigma), 2 µl of 10x T4 RNA ligase buffer (NEB), 1 µl of 40 U/µl RNase Inhibitor (Roche), and 1 µl of T4 ligase 2, truncated (NEB). The ligated products were precipitated and resolved on a 10% TBE-urea gel in 1x TBE at 200V. Using the 10 bp ladder, a band between 45 and 60 bp was excised. After gel extraction, the products were reverse transcribed using Superscript III (Invitrogen) in a 20 µl reaction volume at 50°C for 30 min [[2](#_ENREF_2)] with no more than five molar excess of Reverse transcription primer, 5′-(Phos)-AGATCGGAAGAGCGTCGTGTAGGGAAAGAGTGTAGATCTCGGTGGTCGC-(SpC18)-CACTCA-(SpC18)-TTCAGACGTGTGCTCTTCCGATCTATTGATGGTGC CTACAG-3′. NaOH was added to hydrolyze RNA products were (final concentration of 0.1 mM) and incubated at 95°C C for 15 min. A 10% TBE-urea gel was used to resolve the reverse transcribed cDNA. A band between 165 to 180 bp was excised using 10 bp ladder as the standard. After gel purification, the product was resuspended in 15 µl of 10 mM Tris pH 8.0 and circularized with CircLigase (EPICENTRE) at 60°C for 1 hr. To inactivate the enzyme, it was heated at 80°C for 10 min. The circDNA was PCR amplified as previously described [[2](#_ENREF_2)] with the Forward library PCR primer, 5′-AATGATACGGCGACCACCGAGATCTACAC-3′ and an Indexed reverse library PCR primers, 5′-CAAGCAGAAGACGGCATACGAGATNNNNNN GTGACTG GAGTTCA GACGTGTGCTCTTCCG-3′ (NNNNNN indicates the reverse complement of the index sequence discovered during sequencing. The twelve forward-strand barcode sequences in Table S5). The PCR was done for 7 to 12 cycles using Phusion polymerase (NEB) and the products were resolved on an 8% polyacrylamide gel in 1x TBE at 180V. Using the 10 bp ladder as the standard, a band between 165 to 180 bp was excised. The purified product was resuspended in 10 µl of 10 mM Tris pH 8.0 and quantified by BioAnalyzer (high sensitivity DNA kit, Agilent). The libraries were sequenced on HiSeq2500 platform from Illumina.

*Next Generation Sequencing and alignment to E. coli genome*

The final DNA libraries were validated with Agilent 2100 Bioanalyzer using Agilent High Sensitivity DNA Kit, and the library concentrations were determined by Q-PCR using KAPA SYBRR Fast qPCR kit. The libraries were then run on Single End flowcell on HiSeq 2000 (Illumina) and 52 bp reads were generated. Between 8 to 15 million raw sequence reads were generated for each sample.

All reads were trimmed in a two step process to first remove the adapter sequence “CTGTAGGCACCATCAAT”, discarding reads shorter than 25 nucleotides, and second to remove the first base from every read which was added during the library construction process. This was performed using the FASTX-Toolkit (<http://hannonlab.cshl.edu/fastx_toolkit/>). This trimming process removed an average of 24% of the reads per sample. The remaining 7-11 million reads were aligned using bwa (0.6.2) to the *E. coli* K12 MG1655 reference genome (Genebank version U00096.2) [[4](#_ENREF_4)]. All reads that could not be uniquely mapped to the reference sequence were removed from subsequent analysis, leaving 1.6 – 2.7 million uniquely mapped reads per sample. A TDF file was created for each sample for visualization in IGV, which was scaled to reads per 10 million data using bedtools (2.17.0) [[5](#_ENREF_5)] and igvtools (2.3.3) [[6](#_ENREF_6)]. A coverage file, describing the coverage for each feature in the K12 genome, was created using bedtools. Only ORF that present an average of at least one read per position were considered for further analysis.

For RNA-Seq analysis, coverage was normalized and the means of the WT, ∆*efp* and complemented groups were tested for significant differences using the binomial test in the R package DESeq (1.10.1), producing fold changes and adjusted p-values for each feature [[7](#_ENREF_7)]. Resulting p-values were adjusted for multiple testing with the Benjamin-Hochberg procedure, which controls false discovery rate (FDR).

*Analysis of ribosome densities at PPX sequences and Pausing sites detection.*

Translation pausing sites were searched on the ribosome profiling data using costume analysis scripts written in Perl. In outline, ribosome occupancies were normalized as a ratio of the full ORF average ribosome occupancy. Strong pauses were considered to be segments where the ribosome occupancy was at least 10 fold the gene average [[8](#_ENREF_8)]. Similarly PPX occupancies were normalized as a ratio to the ORF average ribosome occupancies. Strong PPX pauses were considered to have at least 10 fold higher occupancies than the gene average and non pausing PPX were considered to have at most an occupancy equal to the gene average. In addition, non-pausing PPX sequences located just down stream of a translation pause were excluded of the list of non-pausing PPX to prevent including sequences that have low reads because ribosomes were unable to cross a previous point of the mRNA.

*Anti Shine-Dalgarno hybridization free-energy prediction.*

The free-energy of hybridization to anti shine-Dalgarno was estimated with a method similar to what has previously been described [[8](#_ENREF_8)]. A sliding window of 8 nucleotides was selected form each mRNA sequence and the energy of binding to the sequence 5'-CACCUCCU-3' (*E. coli* aSD) was calculated using RNAsubopt program in the Vienna RNA package [[9](#_ENREF_9)] considering a temperature of 37°C. The predicted free-energy was assigned to the position of the last nucleotide in the sliding window.

*Other sequences analysis.*

Alignment of sequences upstream of pausing and non-pausing PPX was performed manually. These alignments were analyzed using Weblogo 3.3 [[10](#_ENREF_10)]. Codon usage for codons in the “X” position of PPX was analyzed using costume Perl scripts, based on codon usage of highly expressed genes from *E. coli* [[11](#_ENREF_11)].

**References**

1. Oh E, Becker AH, Sandikci A, Huber D, Chaba R, et al. (2011) Selective ribosome profiling reveals the cotranslational chaperone action of trigger factor in vivo. Cell 147: 1295-1308.

2. Ingolia NT, Brar GA, Rouskin S, McGeachy AM, Weissman JS (2012) The ribosome profiling strategy for monitoring translation in vivo by deep sequencing of ribosome-protected mRNA fragments. Nat Protoc 7: 1534-1550.

3. Ingolia NT (2010) Genome-wide translational profiling by ribosome footprinting. Methods Enzymol 470: 119-142.

4. Li H, Durbin R (2009) Fast and accurate short read alignment with Burrows-Wheeler transform. Bioinformatics 25: 1754-1760.

5. Quinlan AR, Hall IM (2010) BEDTools: a flexible suite of utilities for comparing genomic features. Bioinformatics 26: 841-842.

6. Thorvaldsdottir H, Robinson JT, Mesirov JP (2013) Integrative Genomics Viewer (IGV): high-performance genomics data visualization and exploration. Brief Bioinform 14: 178-192.

7. Anders S, Huber W (2010) Differential expression analysis for sequence count data. Genome Biol 11: R106.

8. Li GW, Oh E, Weissman JS (2012) The anti-Shine-Dalgarno sequence drives translational pausing and codon choice in bacteria. Nature 484: 538-541.

9. Lorenz R, Bernhart SH, Honer Zu Siederdissen C, Tafer H, Flamm C, et al. (2011) ViennaRNA Package 2.0. Algorithms Mol Biol 6: 26.

10. Crooks GE, Hon G, Chandonia JM, Brenner SE (2004) WebLogo: a sequence logo generator. Genome Res 14: 1188-1190.

11. Thanaraj TA, Argos P (1996) Ribosome-mediated translational pause and protein domain organization. Protein Sci 5: 1594-1612.
